# The Effect of Common Signals on Power, Coherence and Granger Causality: Theoretical Review, Simulations, and Empirical Analysis of Fruit Fly LFPs Data

**DOI:** 10.3389/fnsys.2018.00030

**Published:** 2018-07-25

**Authors:** Dror Cohen, Naotsugu Tsuchiya

**Affiliations:** ^1^School of Psychological Sciences, Monash University, Melbourne, VIC, Australia; ^2^Monash Institute of Cognitive and Clinical Neuroscience, Monash University, Melbourne, VIC, Australia

**Keywords:** common signals, Granger causality, coherence, local field potential (LFP), bipolar signals, unipolar signals

## Abstract

When analyzing neural data it is important to consider the limitations of the particular experimental setup. An enduring issue in the context of electrophysiology is the presence of common signals. For example a non-silent reference electrode adds a common signal across all recorded data and this adversely affects functional and effective connectivity analysis. To address the common signals problem, a number of methods have been proposed, but relatively few detailed investigations have been carried out. As a result, our understanding of how common signals affect neural connectivity estimation is incomplete. For example, little is known about recording preparations involving high spatial-resolution electrodes, used in linear array recordings. We address this gap through a combination of theoretical review, simulations, and empirical analysis of local field potentials recorded from the brains of fruit flies. We demonstrate how a framework that jointly analyzes power, coherence, and quantities based on Granger causality reveals the presence of common signals. We further show that subtracting spatially adjacent signals (bipolar derivations) largely removes the effects of the common signals. However, in some special cases this operation itself introduces a common signal. We also show that Granger causality is adversely affected by common signals and that a quantity referred to as “instantaneous interaction” is increased in the presence of common signals. The theoretical review, simulation, and empirical analysis we present can readily be adapted by others to investigate the nature of the common signals in their data. Our contributions improve our understanding of how common signals affect power, coherence, and Granger causality and will help reduce the misinterpretation of functional and effective connectivity analysis.

## Introduction

Understanding how brain areas communicate is one of the fundamental goals of neuroscience. Such neuroscientific investigations often examine communications between brain areas by assessing functional connectivity or effective connectivity. Here we use the term functional connectivity to refer to correlational (or undirected) relationships and the term effective connective to refer to causal (or directed) relationships as in Bressler and Seth (2010). Functional and effective connectivity are thought to be involved in fundamental processes such as attention (Gregoriou et al., 2009; Bosman et al., 2012; Rosenberg et al., 2016) and arousal (Boveroux et al., 2010; Supp et al., 2011; Lee et al., 2013; Cohen et al., 2016, 2018; Hudetz and Mashour, 2016) and may also be altered in several brain disorders (Horwitz and Horovitz, 2012). Such connectivity analysis may focus on different spatiotemporal scales, from brain-wide connectivity in the order of seconds, as revealed by functional magnetic resonance imaging (Van den Heuvel and Hulshoff Pol, 2010), to micrometer and millisecond resolution in cortical laminar recordings (Maier et al., 2010; Van Kerkoerle et al., 2014).

However, methodological issues regarding the estimation of functional and effective connectivity are a matter of continuing debate (see Barnett et al., 2017; Stokes and Purdon, 2017, for a recent example). An enduring issue in functional and effective connectivity analysis is the adverse effect of common signals in the data. In the context of electrophysiology, the common signals maybe due to electrical activity at the reference electrode, volume conduction from another electrical source, or a combination of both (Bastos and Schoffelen, 2016; Trongnetrpunya et al., 2016). The common signals can substantially alter the results of functional or effective connectivity analysis (Rappelsberger, 1989; Nunez et al., 1997; Essl and Rappelsberger, 1998; Bastos and Schoffelen, 2016; Trongnetrpunya et al., 2016). To this end, numerous techniques of varying complexity have been suggested for reducing the effect of common signals (Nunez et al., 1997; Nolte et al., 2004; Yao et al., 2005; Hu et al., 2010; Brookes et al., 2012; Hipp et al., 2012; Madhu et al., 2012; Drakesmith et al., 2013; Colclough et al., 2015; Huang et al., 2017, and others). One of the simplest and most commonly used techniques is to simply subtract nearby signals, known as bipolar derivations.

Even though the adverse effects of common signals on functional and effective connectivity analysis are well recognized, relatively few studies have investigated these effects in depth. In a recent effort to address this, Shirhatti et al. investigated how different referencing techniques (including bipolar derivations) affect the estimation of different neurophysiological metrics using local field potentials (LFPs) recorded from the visual areas of monkeys (Shirhatti et al., 2016). They found that a measure of functional connectivity known as phase coherence was substantially affected by the choice of the referencing scheme. In particular, they claimed that bipolar derivations can result in artifactually high phase coherence for specific signal pairs. On the other hand, with respect to effective connectivity, Trongnetrpunya et al. used simulations and analysis of LFPs from rats, monkey, and human to claim that bipolar derivations are effective at removing the adverse effect of common signals for Granger causality analysis (Trongnetrpunya et al., 2016). The issue of which referencing scheme is advantageous and disadvantageous in distinct analysis methods remains an open question.

As reviewed above, to date, in-depth studies of the effects of common signals have been carried out separately for functional connectivity (or coherence) and effective connectivity (or Granger causality), not in a unified manner. This may be a significant oversight because coherence and Granger causality are in fact analytically related (Ding et al., 2006; Wen et al., 2013). In this paper, we focus on coherence and Granger causality because these are two of the most commonly used functional and effective connectivity metrics. Through the joint analysis of coherence and Granger causality, we obtain novel theoretical insights into the nature of the common signals and provide empirical guidelines on how to assess and reduce them.

The paper has two major components; “Theoretical background” and “Results from analysis of fly LFPs.” In the Theoretical background sections we review a simple mathematical framework that explains how common signals affect power, coherence and Granger causality. The mathematical framework for power and coherence is based on well-known results from linear dynamics (e.g., Bendat and Piersol, 2000) and has been reported elsewhere (e.g., Rappelsberger, 1989; Nunez et al., 1997; Essl and Rappelsberger, 1998; Yao et al., 2005; Hu et al., 2010; Trongnetrpunya et al., 2016). A more detailed treatment on Granger causality analysis can be found in Ding et al. (2006), Dhamala et al. (2008), Nalatore et al. (2009), Chicharro (2012), Wen et al. (2013). These “Theoretical Background” sections do not contain novel theoretical development, but serve to aggregate these known results in the context of neuroscience in a single manuscript and using unified terminology. We take great care to present the material in the simplest possible way, making it suitable for readers with minimal technical background. Further methodological details are presented in the accompanying Methods section. We complement the theoretical concepts with illustrative simulation designed to clarify the important points. We base our simulations on simple autoregressive processes. Autoregressive processes are the basis of the spectral formulation of Granger causality developed in Geweke (1982, 1984), which we investigate here. For a review of this most widely adopted formulation of Granger causality, see Bressler and Seth (2010). For more recent variants of Granger causality that can deal with non-linear, point-process or otherwise complex dynamics (see e.g., Kim et al., 2011; Quinn et al., 2011; Barnett and Seth, 2014; Sheikhattar et al., 2018). In addition, for autoregressive processes, power and coherence can also be calculated directly from the autoregressive parameters, which further simplifies the simulations. Others have also used autoregressive processes to investigate Granger causality (e.g., Ding et al., 2006; Dhamala et al., 2008; Nalatore et al., 2009; Chicharro, 2012; Wen et al., 2013) and coherence (Rappelsberger, 1989). Codes for running the simulations are made publicly available.

The second component of the paper corresponds to the analysis of LFPs recorded from the brains of flies. The analysis is carried out in parallel to, and immediately following, the relevant Theoretical background sections. This structure highlights the relationship between theory and experiment. We emphasize that the purpose of the analysis is not to suggest a new way for removing common signals, for which a plethora of methods of varying complexity already exists (e.g., Rappelsberger, 1989; Nunez et al., 1997; Essl and Rappelsberger, 1998; Yao et al., 2005; Nalatore et al., 2007, 2009; Hu et al., 2010; Brookes et al., 2012; Madhu et al., 2012; Drakesmith et al., 2013; Faes et al., 2013; Friston et al., 2014; Colclough et al., 2015). Rather, our purpose is to investigate whether substantial common signals are indeed present in our preparation, which involves flies and high-resolution electrodes with spacing in the order of 0.01 mm using a combination of theory, simulation, and empirical analysis.

We show that common signals are present in our preparation and that the bipolar derivations are largely free of these. However, in our theoretical analysis and simulation, we also show that in some special cases the bipolar derivation actually introduces common signals. In addition, we show that a quantity referred to as “instantaneous interaction” is increased in the presence of common signals and may serve as a useful indicator for checking for common signals in other recording preparations. Taken together the manuscript provides a holistic treatment of the effects of common signals on power, coherence and Granger causality using theory, simulation, and empirical analysis.

## Theoretical background and results from analysis of fly LFPs

The theoretical results we present for power and coherence follow from linear dynamics (e.g., Bendat and Piersol, 2000) and have been reported elsewhere (e.g., Rappelsberger, 1989; Nunez et al., 1997; Essl and Rappelsberger, 1998; Yao et al., 2005; Hu et al., 2010; Trongnetrpunya et al., 2016). The theoretical results for Granger causality analysis can be found in Ding et al. (2006), Dhamala et al. (2008), Chicharro (2012), and Wen et al. (2013). Our purpose here is (1) to present these results in a single framework, (2) to illustrate the theoretical concepts using simple simulations, and (3) to demonstrate how these relate to analysis of empirical data. For the analysis of empirical data we use LFPs recording from flies (see Methods for details). We have previously presented analysis results from this dataset in the context of arousal modulation (general anesthesia) (Cohen et al., 2018). In that work we restricted our analysis to bipolar derivations. Here we exclusively focused on the comparison between unipolar signals and bipolar derivations and their relation to the presence of common signals. For neuroscientific insights obtained from this experiment we refer the reader to Cohen et al. (2016, 2018).

### Power: theoretical background

#### Unipolar power

We consider the general case in which the signal recorded by some electrode i at time t *y*_*i*_(*t*) represents the difference of two other signals; the neural activity of interest near the electrode *x*_*i*_(*t*) and a common signal *u*(*t*) that is subtracted from all electrodes

(1) $$\begin{array}{r} y_{i}\left(t\right)=x_{i}\left(t\right)-u\left(t\right) \end{array}$$

We refer to *y*_*i*_(*t*) as a unipolar signal. This framework has been used extensively to investigate the case where the physical mechanisms for the common signal is electrical activity at the reference electrode (i.e., non-silent reference Nunez et al., 1997; Essl and Rappelsberger, 1998; Yao et al., 2005; Hu et al., 2010; Trongnetrpunya et al., 2016). Another mechanism for common signals is volume conduction (Bastos and Schoffelen, 2016; Trongnetrpunya et al., 2016). We consider the distinction between the two mechanisms in the coherence section of this paper (Section Distinguishing Between Common Signals Due to Electrical Activity at the Reference Electrode and Due to Volume Conduction)

In the frequency domain Equation (1) becomes

(2) $$\begin{array}{r} Y_{i}\left(\omega \right)=X_{i}\left(\omega \right)-U\left(\omega \right) \end{array}$$

where*Y*_*i*_(ω), *X*_*i*_(ω) and *U*(ω) are the Fourier transforms of *y*_*i*_(*t*), *x*_*i*_(*t*) and *u*(*t*) respectively.

The power of the unipolar signals (unipolar power) is given by

(3) Sii(ω)=Yi(ω)Yi*(ω)=(Xi(ω)-U(ω))(Xi(ω)-U(ω))*=Xi(ω)Xi*(ω)-2Re(Xi(ω)U*(ω))+U(ω)U*(ω)

where Re(Xi(ω)U*(ω)) denotes taking the real part of the cross-spectrum, also known as the co-spectrum. The co-spectrum captures the effect of zero-lag correlations on the power spectrum. If the common signal is uncorrelated with the neural activity (as may be expected from a noisy common signal from the reference electrode) then the co-spectrum vanishes and the unipolar power is the sum of the power of the neural and common signals

(4) $$\begin{array}{r} S_{ii}\left(\omega \right)=X_{i}\left(\omega \right)X_{i}^{*}\left(\omega \right)+U\left(\omega \right)U^{*}\left(\omega \right) \end{array}$$

In this case the unipolar power is an over-estimate of the power of the neural signal.

#### Bipolar derivations power

The experimenter typically only has access to the unipolar signals *y*_*i*_(*t*), not directly to the neural signal *x*_*i*_(*t*) or the common signal *u*(*t*). The challenge is thus to try and remove, or at least reduce, the contribution of the common signal.

When unipolar activity is simultaneously recorded at two nearby locations one can use the additional signal to reduce the effect of the common signal. The simplest strategy is to take the difference between unipolar signals recorded by nearby electrodes. Specifically, given two nearby unipolar signals *y*_*i*_(*t*) and *y*_*i*+1_(*t*) the bipolar derivation *by*_*i*_(*t*) is defined as

(5) $$\begin{array}{r} by_{i}\left(t\right)=y_{i}\left(t\right)-y_{i+1}\left(t\right) \end{array}$$

If the contribution of the common signal to nearby unipolar signals is identical, then

(6) $$\begin{array}{r} by_{i}\left(t\right)=\left(x_{i}\left(t\right)-u\left(t\right)\right)-\left(x_{i+1}\left(t\right)-u\left(t\right)\right)\\ =x_{i}\left(t\right)-x_{i+1}\left(t\right) \end{array}$$

Thus, bipolar derivations can provide an estimate of local neural activity that is free from the effect of the common signal.

In the frequency domain Equation (6) becomes

(7) $$\begin{array}{r} bY_{i}\left(\omega \right)=X_{i}\left(\omega \right)-X_{i+1}\left(\omega \right) \end{array}$$

The power of the bipolar derivations (bipolar power) is given by

(8) $$\begin{array}{r} bS_{ii}\left(\omega \right)=bY_{i}\left(\omega \right)bY_{i}^{*}\left(\omega \right)=\left(X_{i}\left(\omega \right)-X_{i+1}\left(\omega \right)\right){\left(X_{i}\left(\omega \right)-X_{i+1}\left(\omega \right)\right)}^{*}\\ =X_{i}\left(\omega \right)X_{i}^{*}\left(\omega \right)-2Re\left(X_{i}\left(\omega \right)X_{i+1}^{*}\left(\omega \right)\right)+X_{i+1}\left(\omega \right)X_{i+1}^{*}\left(\omega \right) \end{array}$$

It is useful to compare the expression for the unipolar (Equation 3) and bipolar power (Equation 8). In general, to completely characterize the unipolar (*S*_*ii*_(ω)) and bipolar (*bS*_*ii*_(ω)) power, we would need to know (1) the power of neural signals ($X_{i}\left(\omega \right)X_{i}^{*}\left(\omega \right)$ and $X_{i+1}\left(\omega \right)X_{i+1}^{*}\left(\omega \right)$), (2) the power of the common signal (*U*(ω)U^*^(ω)), and (3) the relevant co-spectra [$Re\left(X_{i}\left(\omega \right)X_{i+1}^{*}\left(\omega \right)\right)$ and $Re\left(X_{i}\left(\omega \right)U^{*}\left(\omega \right)\right)$]. For example, assuming that the co-spectra are negligible in both cases, a stronger common signal [*U*(ω)U^*^(ω)] than the power of neural signals [$X_{i+1}\left(\omega \right)X_{i+1}^{*}\left(\omega \right)$] can result in greater unipolar than bipolar power. Another interesting case is when the neural signals recorded by nearby electrodes are very similar, [i.e., *X*_*i*_(ω)~*X*_*i*+1_(ω)]. In this case, the bipolar power is obviously close to zero, while the unipolar power can be much greater than zero.

In the context of neurophysiology, both scenarios are possible (e.g., substantial non-silent reference, highly similar recordings by nearby electrodes). Under these circumstances we would expect the unipolar signals power to be greater than the bipolar derivations power. Theoretically speaking, however, it is entirely possible for the bipolar power to be *greater* than the unipolar power. For example if the power of the neural signals is equal, $X_{i+1}\left(\omega \right)X_{i+1}^{*}\left(\omega \right)=X_{i}\left(\omega \right)X_{i}^{*}\left(\omega \right)=P$ and if the neural signals are independent Re(Xi(ω)Xi+1*(ω))=0, then the power of the bipolar derivation is *bS*_*ii*_(ω) = 2*P*. If the common signal is comparatively small, e.g., *U*(ω)U^*^(ω) = *P*/2, then the power of the unipolar signals is $S_{ii}\left(\omega \right)=P+\frac{P}{2}$.

### Power: results from analysis of fly LFPs

#### Unipolar power is greater than bipolar power

**Figure 1:**
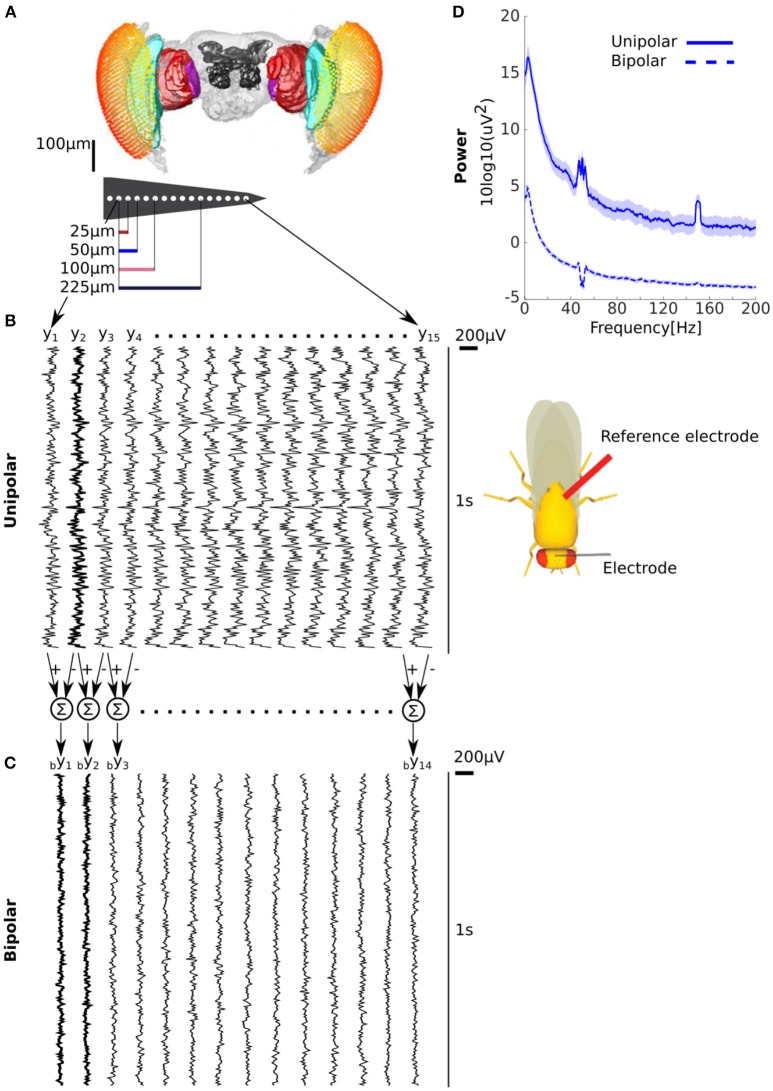
Unipolar power is greater than bipolar power in fly LFP. **(A)** Microelectrode recordings of LFPs from flies. Adjacent electrodes are separated by 25 μm. **(B)** Example of unipolar signals from one fly. The unipolar signals were recorded with reference to the thorax (location shown on schematic on the right). The example clearly shows that the signals are highly correlated across electrodes but that any neighboring pairs are not the same. **(C)** Example of bipolar derivations obtained from two adjacent unipolar signals. The bipolar derivations are smaller in magnitude and appear less correlated than the unipolar signals. Two bipolar derivations (by1 and by2) that share a unipolar signal (y2 in **B**) in their derivation are shown in bold. **(D)** Group average unipolar (solid) and bipolar (dashed) power (*N* = 13, shading reflects standard error of the mean across flies). The much greater unipolar than bipolar power is consistent with substantial common signals and the highly similar neural activity of adjacent unipolar signals. Peaks at 50 and 150 Hz reflect line noise and its harmonic.

The mathematical framework we presented demonstrates that in the presence of a common signal the unipolar power can be either greater or lesser than the bipolar power. Next we empirically quantified unipolar and bipolar power for LFPs recorded with a micro-electrode linear array inserted in the brains of behaving flies (Figure 1, see Methods for details). We considered signals recorded with respect to a reference electrode inserted in the body (thorax) of the fly as unipolar (Figure 1B). We obtained bipolar derivations by subtracting adjacent unipolar signals (Figure 1C, see Methods for details). In our data we expected unipolar to be greater than bipolar power because (1) there is likely to be electrical activity at the reference electrode in the flies' thorax (2) neighboring electrodes are likely to reflect at least some similar neural activity due to the proximity of the electrodes (i.e., 25 μm). Consistent with this we found that unipolar was indeed greater than the bipolar power (Figure 1D).

### Coherence: theoretical background

#### Unipolar coherence

Coherence between signals*Y*_*i*_(ω) and*Y*_*j*_(ω) measures the extent of linear dependency at each frequency (Bendat and Piersol, 2000) and is defined as:

(9) $$\begin{array}{r} C_{ij}\left(\omega \right)=\frac{abs{\left(Y_{i}\left(\omega \right)Y_{j}^{*}\left(\omega \right)\right)}^{2}}{\left(Y_{i}\left(\omega \right)Y_{i}^{*}\left(\omega \right)\right)\left(Y_{j}\left(\omega \right)Y_{j}^{*}\left(\omega \right)\right)} \end{array}$$

As we will show, in the presence of a common signal, coherence can be non-zero even when the neurophysiological signals [*X*_*i*_(ω) and *X*_*j*_(ω)] themselves are independent. To see the effect of a common signal on unipolar signals coherence we substitute Equation (2) into (9), obtaining:

(10) $$\begin{array}{r} C_{ij}\left(\omega \right)=\frac{abs{\left(\left(X_{i}\left(\omega \right)-U\left(\omega \right)\right){\left(X_{j}\left(\omega \right)-U\left(\omega \right)\right)}^{*}\right)}^{2}}{\left(X_{i}\left(\omega \right)-U\left(\omega \right)\right){\left(X_{i}\left(\omega \right)-U\left(\omega \right)\right)}^{*}\left(X_{j}\left(\omega \right)-U\left(\omega \right)\right){\left(X_{j}\left(\omega \right)-U\left(\omega \right)\right)}^{*}} \end{array}$$

If the common signal and neural signals are independent then the cross-spectrum between the two vanishes and Equation (10) becomes

(11) $$\begin{array}{r} C_{ij}\left(\omega \right)=\frac{abs{\left(X_{i}\left(\omega \right)X_{j}^{*}\left(\omega \right)+U\left(\omega \right)U^{*}\left(\omega \right)\right)}^{2}}{\left(X_{i}\left(\omega \right)X_{i}^{*}\left(\omega \right)+U\left(\omega \right)U^{*}\left(\omega \right)\right)\left(X_{j}\left(\omega \right)X_{j}^{*}\left(\omega \right)+U\left(\omega \right)U^{*}\left(\omega \right)\right)} \end{array}$$

Because we do not have direct access to each of the individual quantities we cannot in general assess the contribution of the common signal to coherence. Here we suggest using prior knowledge of the system in question to try and isolate the relative contribution of the common signal. For example, for neural systems we would not expect genuine neurophysiological coupling for very high frequencies. That is, for high frequencies ω_*h*_ we assume that $X_{i}\left(\omega_{h}\right)X_{j}^{*}\left(\omega_{h}\right)=0$. In this case Equation (11) becomes

(12) $$\begin{array}{r} C_{ij}\left(\omega \right)=\frac{abs{\left(U\left(\omega_{h}\right)U^{*}\left(\omega_{h}\right)\right)}^{2}}{\left(X_{i}\left(\omega_{h}\right)X_{i}^{*}\left(\omega_{h}\right)+U\left(\omega_{h}\right)U^{*}\left(\omega_{h}\right)\right)\left(X_{j}\left(\omega_{h}\right)X_{j}^{*}\left(\omega_{h}\right)+U\left(\omega_{h}\right)U^{*}\left(\omega_{h}\right)\right)} \end{array}$$

This shows that a common signal can render a non-zero coherence even when the neurophysiological sources themselves are not coherent at all.

Observing (implausibly) high coherence in (implausibly) high frequencies would indicate that a common signal is present, and is likely to affect all frequencies [as described by Equation (11)]. Having observed high coherence in high frequencies in a given dataset would therefore warrant extra care when interpreting coherence in lower frequencies, for which there may be some genuine neurophysiological coupling. We also note that what constitutes such “high frequencies” depends on the system in question. For example coherence at frequencies >100 Hz has been reported in mammals (see (Buzsáki and Schomburg, 2015), for discussion of high frequency coherence). Whether these reports were affected by the presence of common signals is unknown. To date, coherence above 100 Hz has not been reported in flies.

Note, however, that as per Equation (12), if the (squared) power of the common signal (numerator) is much smaller than the (product of the) sums of the power of neural signals and common signal (denominator), then we would obtain close to zero coherence. Thus, the absence of high coherence in high frequencies could also be consistent with a common signal that only has low frequency power.

Equation (12) can be further simplified. First, we define the Neural to Common signal Ratio as $NCR_{i}\left(\omega_{h}\right)=\frac{X_{i}\left(\omega_{h}\right)X_{i}^{*}\left(\omega_{h}\right)}{U\left(\omega_{h}\right)U^{*}\left(\omega_{h}\right)}$

Second, assuming that the NCR is comparable across all electrodes (*NCR*_*i*_(ω_*h*_)~ *NCR*_*j*_(ω_*h*_) = *NCR*(ω_*h*_)) we obtain coherence as a function of a single parameter, *NCR*(ω_*h*_),

(13) $$\begin{array}{r} C_{ij}\left(\omega_{h}\right)=\frac{1}{{\left(NCR\left(\omega_{h}\right)+1\right)}^{2}} \end{array}$$

Using Equation (13) we can get an estimate of the NCR only using the observed coherence values

(14) $$\begin{array}{r} NCR\left(\omega_{h}\right)=\frac{1}{\sqrt{C_{ij}\left(\omega_{h}\right)}}-1 \end{array}$$

#### Distinguishing between common signals due to electrical activity at the reference electrode and due to volume conduction

The simple theoretical framework we reviewed assumes that a single common signal u(t) enters *all electrodes equally*. This situation most directly reflects a common signal due to electrical activity at the reference electrode, as this is subtracted from all channels. Another mechanism for common signals is the conduction of electrical activity from some other electrical source to two or more electrodes, known as volume conduction (Bastos and Schoffelen, 2016). One potential source for such electrical activity is muscular activity. For example, in human scalp and intracranial EEG, eye movements and blinks generate electrical activity that maybe conducted through the scalp or neural tissue and manifest as a common signal (Kovach et al., 2011). In the context of our fly preparation, the non-neuronal electrical activity may be due to the heart muscle (Paulk et al., 2013; Yap et al., 2017). The difference between the common signal from the electrical activity at the reference and common signals due to volume conduction is that the latter are likely to affect nearby electrodes more than electrodes that are far apart, since the contribution from volume conduction decays with increasing distance (Nunez et al., 1997). This spatial-dependence would violate our assumption that the same common signal affects all electrodes. A general way to include a distance-dependent effect such as that from volume conduction into the framework is to decompose the common signals contribution *u*(*t*) into the difference of a “global” common signal *r*(*t*), reflecting activity at the reference electrode, and an electrode-dependent signal *vc*_*i*_(*t*), reflecting common signals due to Volume Conduction. The resulting expression for the unipolar signals is

(15) $$\begin{array}{r} y_{i}\left(t\right)=x_{i}\left(t\right)-u_{i}\left(t\right)\\ =x_{i}\left(t\right)+vc_{i}\left(t\right)-r\left(t\right) \end{array}$$

Then, coherence is given by

(16) $$\begin{array}{r} C_{ij}\left(\omega \right)=\frac{abs{\left(\left(X_{i}\left(\omega \right)+VC_{i}\left(\omega \right)-R\left(\omega \right)\right){\left(X_{j}\left(\omega \right)+VC_{j}\left(\omega \right)-R\left(\omega \right)\right)}^{*}\right)}^{2}}{\left(X_{i}\left(\omega \right)+VC_{i}\left(\omega \right)-R\left(\omega \right)\right){\left(X_{i}\left(\omega \right)+VC_{i}\left(\omega \right)-R\left(\omega \right)\right)}^{*}\left(X_{j}\left(\omega \right)+VC_{j}\left(\omega \right)-R\left(\omega \right)\right){\left(X_{j}\left(\omega \right)+VC_{j}\left(\omega \right)-R\left(\omega \right)\right)}^{*}} \end{array}$$

Unfortunately, even if we make the same two simplifying assumptions as before, namely that the common signal from the reference electrode [*r*(*t*)] is independent of the neural signals (*x*_*i*_(*t*) and *x*_*j*_(*t*)) and that the neural signals themselves are independent for high frequencies, then the expression for coherence remains complicated (see Appendix A in Supplementary Materials).

Using more advanced analysis it may be possible to mathematically estimate the distance-dependent effect in neuronal data, though this is beyond the scope of our current paper. For the purpose of our paper, the qualitative prediction from these theoretical considerations is that in the presence of volume conduction coherence in high frequencies will decrease with increasing separation between the signals (see Appendix A in Supplementary Materials).

#### Bipolar coherence

We can investigate the effects of bipolar derivation on coherence in a similar way to the unipolar signals. Substituting Equation (7) into Equation (9) we get the following expression for coherence between bipolar derivations (bipolar coherence) *bC*_*ij*_(ω)

(17) bCij(ω)=abs(bYi(ω)bYj*(ω))2bYi(ω)bYi*(ω)bYj(ω)bYj*(ω)=abs((Xi(ω)-Xi+1(ω))(Xj(ω)-Xj+1(ω))*)2(Xi(ω)-Xi+1(ω))(Xi(ω)-Xi+1(ω))*(Xj(ω)-Xj+1(ω))(Xj(ω)-Xj+1(ω))*=abs(Xi(ω)Xj*(ω)-Xi(ω)Xj+1*(ω)-Xi+1(ω)Xj*(ω)+Xi+1(ω)Xj+1*(ω))2(Xi(ω)Xi*(ω)-2Re(Xi(ω)Xi+1*(ω))+Xi+1(ω)Xi+1*(ω))(Xj(ω)Xj*(ω)-2Re(Xj(ω)Xj+1*(ω))+Xj+1(ω)Xj+1*(ω))

Note that if all combinations of the neural signals are independent [i.e., when *i* ≠ *j*, $X_{i}\left(\omega \right)X_{j}^{*}\left(\omega \right)=X_{i}\left(\omega \right)X_{j+1}^{*}\left(\omega \right)=X_{i+1}\left(\omega \right)X_{j}^{*}\left(\omega \right)=X_{i+1}\left(\omega \right)X_{j+1}^{*}\left(\omega \right)=0$]then the cross-spectra in the numerator all vanish and coherence will equal zero. This is in contrast to unipolar coherence, which can be above zero even if the neural signals are independent (Equation 12).

Coherence between bipolar derivations that are obtained from a shared unipolar signal (Figure 1C) *bC*_*i,i*+1_(ω) constitutes a special case,

(18) $$\begin{array}{r} bC_{i,i+1}\left(\omega \right)=\frac{abs{\left(\left(X_{i}\left(\omega \right)-X_{i+1}\left(\omega \right)\right){\left(X_{i+1}\left(\omega \right)-X_{i+2}\left(\omega \right)\right)}^{*}\right)}^{2}}{\left(X_{i}\left(\omega \right)-X_{i+1}\left(\omega \right)\right){\left(X_{i}\left(\omega \right)-X_{i+1}\left(\omega \right)\right)}^{*}\left(X_{i+1}\left(\omega \right)-X_{i+2}\left(\omega \right)\right){\left(X_{i+1}\left(\omega \right)-X_{i+2}\left(\omega \right)\right)}^{*}} \end{array}$$

By noting that coherence is invariant with respect to scalar multiplication of the signals, we can use *bY*_*i*+1_(ω) → −*bY*_*i*+1_(ω) = *X*_*i*+2_(ω)−*X*_*i*+1_(ω). Equation (18) becomes

(19) bCi,i+1(ω)=abs((Xi(ω)-Xi+1(ω))(Xi+2(ω)-Xi+1(ω))*)2(Xi(ω)-Xi+1(ω))(Xi(ω)-Xi+1(ω))*(Xi+2(ω)-Xi+1(ω))(Xi+2(ω)-Xi+1(ω))*=abs(Xi(ω)Xi+2*(ω)-Xi(ω)Xi+1*(ω)-Xi+1(ω)Xi+2*(ω)+Xi+1(ω)Xi+1*(ω))2(Xi(ω)Xi*(ω)-2Re(Xi(ω)Xi+1*(ω))+Xi+1(ω)Xi+1*(ω))(Xi+1(ω)Xi+1*(ω)-2Re(Xi+1(ω)Xi+2*(ω))+Xi+2(ω)Xi+2*(ω))

This expression is identical to the case of unipolar coherence (Equation 10) where the common signal *U*(ω) has been replaced with the neural signal *X*_*i*+1_(ω). Intuitively, the shared signal *X*_*i*+1_(ω) acts as a common signal.

If the signals *X*_*i*_(ω_*h*_), *X*_*i*+1_(ω_*h*_) and *X*_*i*+2_(ω_*h*_) are independent for high frequencies ω_*h*_, then

$$\begin{array}{l} b{C_{i,}}_{i+1}\left(\omega_{h}\right)=\frac{abs{\left(X_{i+1}\left(\omega_{h}\right)X_{i+1}^{*}\left(\omega_{h}\right)\right)}^{2}}{\left(X_{i}\left(\omega_{h}\right)X_{i}^{*}\left(\omega_{h}\right)+X_{i+1}\left(\omega_{h}\right)X_{i+1}^{*}\left(\omega_{h}\right)\right)\left(X_{i+2}\left(\omega_{h}\right)X_{i+2}^{*}\left(\omega_{h}\right)+X_{i+1}\left(\omega_{h}\right)X_{i+1}^{*}\left(\omega_{h}\right)\right)} \end{array}$$

Thus, coherence between bipolar derivations that share a unipolar signal in their derivation can be above zero even if neural activity is independent. For example, if the power of the neural activity is equal across channels i, i + 1 and i + 2 [i.e., $X_{i}\left(\omega_{h}\right)X_{i}^{*}\left(\omega_{h}\right)=X_{i+1}\left(\omega_{h}\right)X_{i+1}^{*}\left(\omega_{h}\right)=X_{i+2}\left(\omega_{h}\right)X_{i+2}^{*}\left(\omega_{h}\right)$] then *b*_*C*_*i*_, *i*+1_(ω_*h*_) = 0.25.

### Example simulations: the effect of a common signal on a unidirectionally-connected and a disconnected system

**Figure 2:**
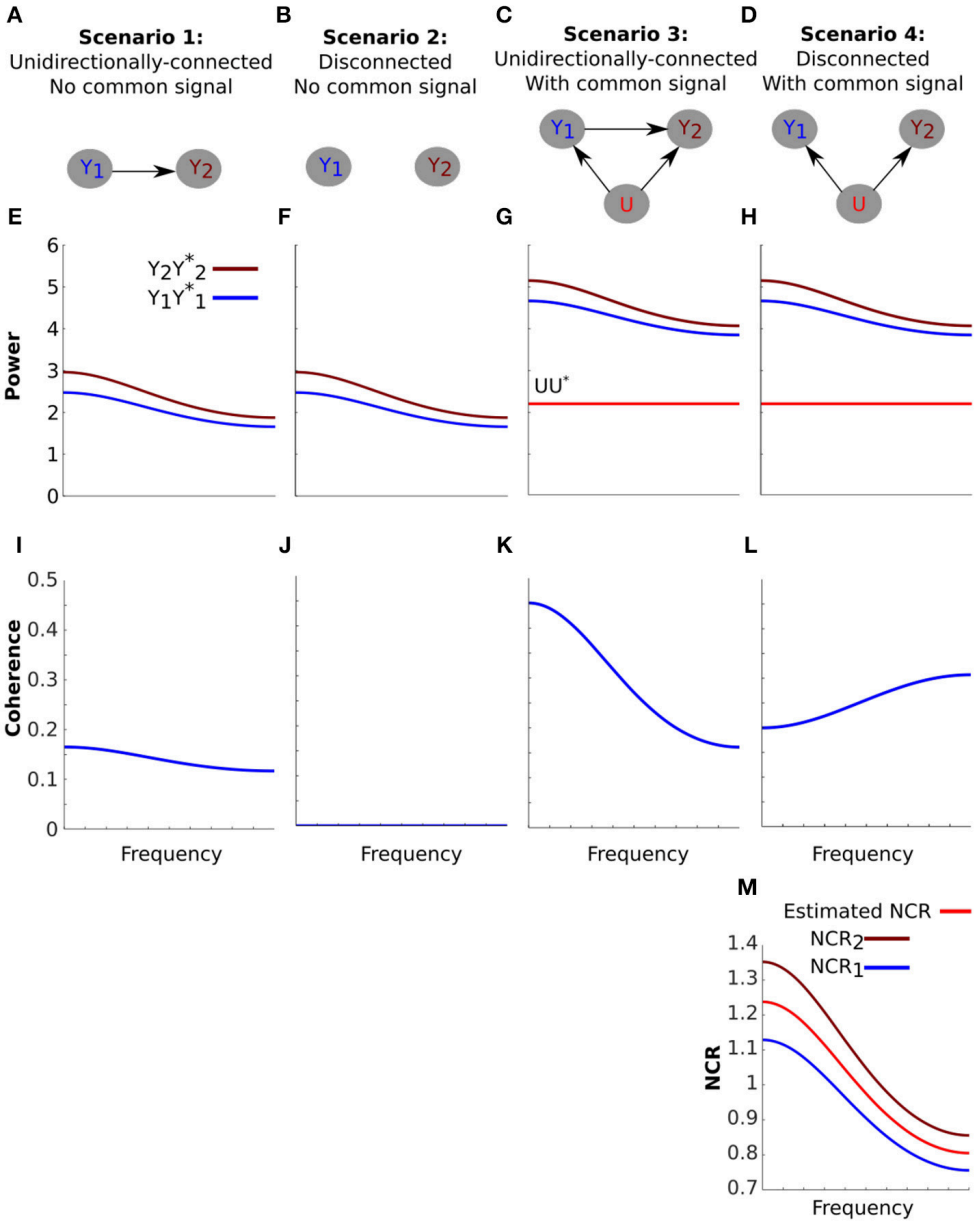
The effect of a common signal on coherence is highly dependent on the spectral characteristics of the system. **(A-D)** Schematics of four simulated scenarios corresponding to a unidirectionally-connected system without a common signal **(A)**, a disconnected system without a common signal **(B)**, a unidirectionally-connected system with a common signal **(C)** and a disconnected system with a common signal **(D)**. **(E–H)** The power of the signals for each scenario (arbitrary units). The systems were constructed to have identical power spectra (**E** vs. **F** and **G** vs. **H**). The effect of the common signal on power **(G,H)** is an increase equal to the power of the common signal (UU*, horizontal red line). **(I–L)** Coherence between the signals for each scenario. Coherence for the unidirectionally-connected system is low and decays with frequency **(I)** and coherence for the disconnected system is zero **(J)**. When a common signal is added to the unidirectionally-connected system coherence values increase overall but still decay with frequency **(K)**. When a common signal is introduced to the disconnected system coherence values also increase, but now coherence increases with frequency **(L)**. This unexpected increase is explained by the Neural to Common signal Ratio (NCR) of this particular system (Equation 13). **(M)** NCR1 (blue) and NCR2 (brown) refer to the NCR of nodes one and two respectively. Because the NCRs for this system decrease with frequency, coherence increases with frequency. From the observed coherence in **(L)**, we can also estimate the NCR using Equation (14). The estimated NCR is shown here in red, which falls between the value of NCR1 and NCR2.

The theoretical framework describes how a common signal can affect coherence. We next use simple simulations to illustrate how the presence of a common signal may manifest in the analysis of real data. To do this we considered four scenarios (Figures 2A–D). Scenarios 1 and 2 correspond to a unidirectionally-connected and a disconnected neural system (meaning that the cross-spectrum between the signals is zero for all frequencies). These two scenarios represent the “ground truths”. Scenarios 3 and 4 represent the same two systems but in the presence of a common signal that is uncorrelated with both components of the system.

We modeled the unidirectionally-connected system as a bivariate autoregressive process (see Methods for details)

(20) $$\begin{array}{r} y_{1}\left(t\right)=ay_{1}\left(t-1\right)+\eta \left(t\right)\\ y_{2}\left(t\right)=cy_{2}\left(t-1\right)+dy_{1}\left(t-1\right)+\varepsilon \left(t\right) \end{array}$$

where a, c, and d are the autoregressive coefficients and ε(*t*) and η(*t*) represent uncorrelated white Gaussian noise sources. For autoregressive systems such as this, power, coherence, and Granger causality can all be directly calculated from the autoregressive parameters (see Methods for details).

We set the parameters of the autoregressive process such that the power spectra of the unidirectionally connected system decays with frequency, roughly reflecting a biological system (Figure 2E). We intentionally constructed the disconnected system such that its power spectrum is identical to the unidirectionally-connected system (Figure 2F, see Methods for details). Thus, the two systems are indistinguishable based on their power spectra alone.

The effect of the uncorrelated common signal on power is given by Equation (4). This effect corresponds to an increase in power equal to the power of the common signal. Note that Equation (4) is independent of the connectivity of the system. This means that *the effect* of the common signal on power is identical for the unidirectionally-connected (Figure 2G) and disconnected systems (Figure 2H). Thus, power analysis alone cannot distinguish the two systems.

Next we assessed coherence. The parameters of the autoregressive process result in low coherence values that decay with frequency for the unidirectionally-connected system (Figure 2I). For the disconnected system coherence is zero, as expected (Figure 2J). The common signal increases coherence for the unidirectionally-connected system (more so for lower frequencies) (Figure 2K). Importantly, the common signal introduces above-zero coherence even for the disconnected system (Figure 2L). For both the connected and disconnected systems (Figures 2K,L), this simulation clearly demonstrates that a common signal can lead to an overestimation of coherence.

Note that in the disconnected system (Figure 2L) the common signal *increased* coherence with *frequency*. This frequency-dependent increase would be hard to understand without the theoretical framework. However, our theoretical framework actually explains it as a consequence of our choice of parameters. Specifically, Equation (13) dictates that coherence is inversely related to Neural Signal to Common signal Ratios (NCRs). In our disconnected system, the NCRs of both signals decrease with frequency (as a consequence of the power spectra that decays with increasing frequency). In such a case, Equation (13) explains that coherence should increase with frequency, as we see here.

In fact, if we assume that the NCR of both signals are identical then we can *use* coherence to estimate the NCR (Equation 14). In our disconnected system, the NCRs of both signals are similar, thus the assumption roughly holds and the resulting estimate is meaningful (Figure 2M).

In sum, our simulations demonstrate how the mathematical framework we presented may manifest in empirical analysis. We however highlight that the effects of a common signal on coherence are dependent on the specific spectral characteristics of the system and common signal. (See Appendix B in Supplementary Materials for a simulation using a common signal with a 1/f-like spectrum instead of a uniform spectrum).

### Coherence: results from analysis of fly LFPs

#### LFPs recorded in flies show very high coherence with unipolar referencing

Based on the theoretical framework and as demonstrated in our simulation (Figure 2), we expect that coherence between unipolar signals, recorded from biological brains, to present the following characteristics. First, we do not expect to find significant coherence in high frequencies [to our best knowledge, coherence for LFPs in flies has not been reported for frequencies greater than 100 Hz (Van Swinderen and Greenspan, 2003; Paulk et al., 2013, 2015; Cohen et al., 2016)]. Conversely, high coherence in high frequencies indicates the presence of common signals. Second, common signals would exert stronger influence on coherence between unipolar signals recorded by closer pairs of electrodes than those that are far apart (due to volume conduction in addition to electrical activity at the reference electrode).

**Figure 3:**
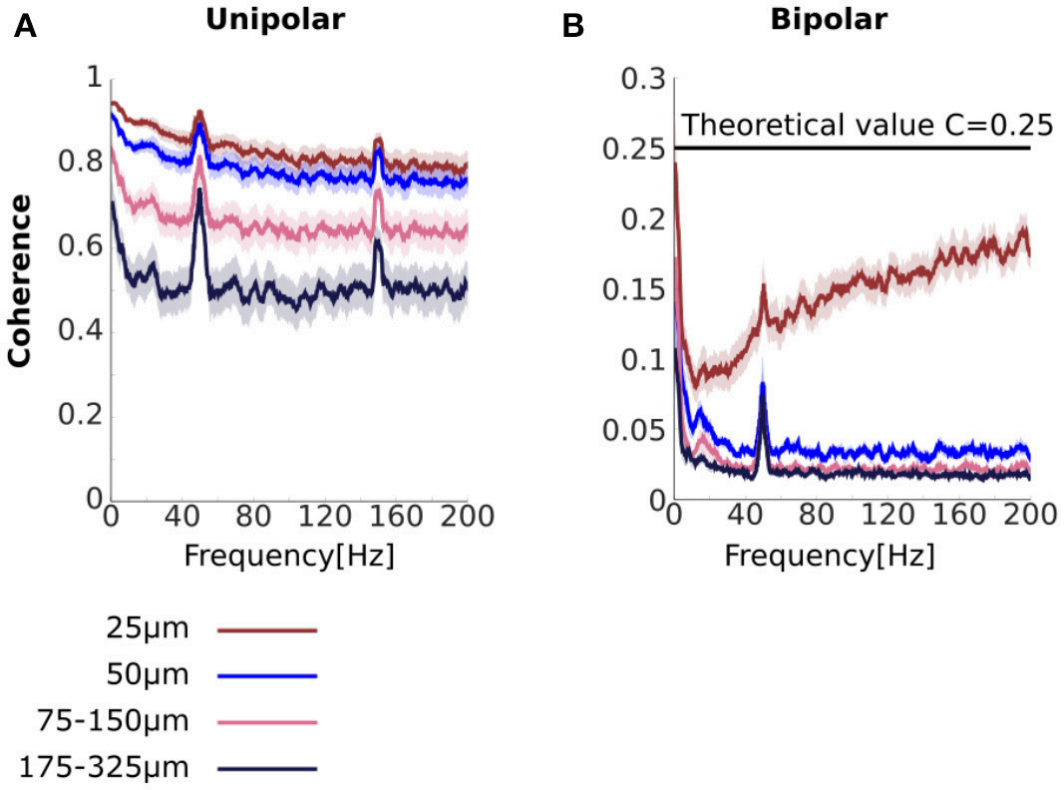
Unipolar and bipolar coherence. **(A,B**) Unipolar **(A)** and bipolar **(B)** coherence averaged across signal pairs separated by 25 (brown), 50 (blue), 75–150 (pink), 175–325 μm (black) (see Methods for details). The very high unipolar coherences are consistent with the presence of strong common signals. Bipolar derivations that share a unipolar signals in their derivation are separated by 25 μm (see Figure 1). Horizontal line at *C* = 0.25 represents theoretical coherence value when the NCR = 1. Peaks at 50 and 150 Hz are due to line noise and its harmonic. Shaded area represents sem across flies (*N* = 13).

We analyzed coherence as a function of the distance between the unipolar signals. To do this we averaged coherence over unipolar signal pairs separated by 25, 50, 75–150, and 175–325 μm (Figure 3A). We found high coherence values across all unipolar pairs. Coherence values were near unity for adjacent unipolar signals (separated by 25 μm) in low frequencies. For these pairs, coherence values decreased with frequency before plateauing at very high values of around 0.8. These very high coherence values in high frequencies in which we would not expect genuine neurophysiological coupling indicate the presence of common signals, which are likely to be affecting all frequencies.

We further found that coherence between unipolar signals decreased with increasing distance between the signals. In low frequencies this reduction may reflect a genuine reduction in neurophysiological coupling with increasing distance. However, the reduction in coherence with distance between the signals was also observed for high frequencies and thus likely to reflect, at least in part, a reduction in the influence of the common signals. This suggests that the properties of the common signals are not constant across all electrodes, consistent with common signals due to volume conduction (see Section Distinguishing between common signals due to electrical activity at the reference electrode and due to volume conduction and Appendix A in Supplementary Materials).

Unipolar signal pairs that are far apart (175–325 μm) are less likely to be affected by volume conduction. However, we found that coherence values were still around 0.5 for these far apart pairs, even for high frequencies for which we would not expect genuine neurophysiological coupling. A likely reason for this high coherence for signals that are far apart is a common signal due to electrical activity at the reference electrode. Under some further simplifying assumptions we can use the coherence values to estimate the Neural signal to Common signal Ratio (see Equation 14 and Figure 2M). Under these assumptions, coherence values of 0.5 translate to an NCR of approximately 0.41, which means that neural activity is less than half of the magnitude of the common signal. Thus, the coherence analysis strongly indicates the presence of a substantial common signal due to electrical activity at the reference electrode located in the flies' thorax (Figure 1B).

#### Coherence between bipolar derivations is low, as per the theoretical prediction, but coherence between adjacent pairs increases with frequency

Our analyses so far (both power and coherence) strongly indicate that the unipolar signals contain substantial common signals. According to the mathematical framework, bipolar derivations can reduce the effect of common signals on coherence (see section Bipolar coherence and Appendix A in Supplementary Materials). With bipolar derivations, we would expect lower coherence values overall and, in particular, coherence would be near zero for very high frequencies for which we do not expect genuine neurophysiological coupling.

To test this, we repeated the coherence analysis using the bipolar derivations (Figure 3B). We found that bipolar coherence was indeed much lower than unipolar coherence. For bipolar derivations separated by 50–325 μm coherence values for low frequencies were in the range 0.025–0.15. Crucially, for higher frequencies we observed near-zero coherence. Taken together these observations suggest that bipolar derivations are largely free from common signals. Bipolar coherence also decreased as the distance between the bipolar derivations increased, potentially indicating a reduction in neurophysiological coupling with increasing distance.

Further, coherence between bipolar derivations separated by 25 μm actually increased with frequency. However, the mathematical framework we provided can fully account for this apparently surprising finding. Bipolar derivations separated by 25 μm are derived from a shared unipolar signal, whereas bipolar derivations separated by 50 μm or more are derived from distinct unipolar signals (Figures 1B,C). Coherence between bipolar derivations that share a unipolar signal in their derivation constitutes a special case, in which the shared unipolar signal effectively acts as a common signal (Equation 19). Coherence in the presence of a common signal can be above zero even if the neural signals themselves are independent.

Recall, further, that coherence also increased with frequency in our simulation of a disconnected system in the presence of a common signal (Figure 2L). In that case, we explained it in terms of the analytical relationship between coherence and the NCRs (Figure 2M). We note here that if we assume that the power of the neural signals is equal, then the NCRs equal 1 and coherence equals 0.25 (Equation 13). As can be seen, coherence approached but never reached this value (Figure 3B, horizontal a black line in), potentially indicating that some assumptions, for example that the power of the neural signals is equal, are not completely satisfied.

### Granger causality: theoretical background

Here we examine how the presence of a common signal can be incorporated into the Granger causality framework. The mathematical specifics of Granger causality have been discussed extensively (e.g. Geweke, 1982, 1984; Lütkepohl, 2005; Dhamala et al., 2008; Chicharro, 2012; Wen et al., 2013; Wibral et al., 2014; Seth et al., 2015). We cover the relevant details to our analysis in the Methods section. More complete details can be found in the literature above and citations therein.

In the context of our analysis, the key analytical result is the relationship

(21) $$\begin{array}{r} -\ln\;\left(1-C_{ij}\left(\omega \right)\right)=f_{i↔j}\left(\omega \right)+f_{i\cdot j}\left(\omega \right) \end{array}$$

where *f*_*i*↔*j*_(ω) represent the sum of Granger causal influences from i to j and j to i, termed total Granger causality, and *f*_*i*·*j*_(ω) represents zero-lag or instantaneous effect, termed instantaneous interaction. This result demonstrates that a simple transformation of coherence [left side of (21)] can be decomposed into total Granger causality [first term on the right side (21)], which capture lagged influences, and instantaneous interaction [second term on the right side of (21)], which captures any remaining instantaneous influences, possibly due to exogenous sources (Ding et al., 2006; Wen et al., 2013; Bastos and Schoffelen, 2016; Trongnetrpunya et al., 2016). We acknowledge concerns about the interpretability of instantaneous interaction since it can become negative in certain situations (Ding et al., 2006; Chicharro, 2012; Wen et al., 2013). However, this quantity may still be useful for empirical analysis. In particular, instantaneous interaction may be increased in the presence of common signals, as we describe below.

The frequency domain representation of the unipolar signals under the Granger causality framework is

(22) $$\begin{array}{r} \left(\begin{array}{c} Y_{i}\left(\omega \right)\\ Y_{j}\left(\omega \right) \end{array}\right)=\left(\begin{array}{cc} H_{ii}\left(\omega \right) & H_{ij}\left(\omega \right)\\ H_{ji}\left(\omega \right) & H_{jj}\left(\omega \right) \end{array}\right)\left(\begin{array}{c} \varepsilon \left(\omega \right)\\ \eta \left(\omega \right) \end{array}\right) \end{array}$$

where $\left(\begin{array}{cc} H_{ii}(\omega ) & H_{ij}(\omega )\\ H_{ji}(\omega ) & H_{jj}(\omega ) \end{array}\right)$ is the transfer function representation of the autoregressive process and $\left(\begin{array}{c} \varepsilon (\omega )\\ \eta (\omega ) \end{array}\right)$ represents the Fourier transforms of the noise terms (see Methods for details).

Combining the frequency domain representation with the expression of the unipolar signals (2) gives

$$\begin{array}{l} \left(\begin{array}{c} X_{i}\left(\omega \right)\\ X_{j}\left(\omega \right) \end{array}\right)-\left(\begin{array}{c} U\left(\omega \right)\\ U\left(\omega \right) \end{array}\right)=\left(\begin{array}{cc} H_{ii}\left(\omega \right) & H_{ij}\left(\omega \right)\\ H_{ji}\left(\omega \right) & H_{jj}\left(\omega \right) \end{array}\right)\left(\begin{array}{c} \varepsilon \left(\omega \right)\\ \eta \left(\omega \right) \end{array}\right) \end{array}$$

In terms of the neural signals we get

(23) $$\begin{array}{r} \left(\begin{array}{c} X_{i}\left(\omega \right)\\ X_{j}\left(\omega \right) \end{array}\right)=\left(\begin{array}{cc} H_{ii}\left(\omega \right) & H_{ij}\left(\omega \right)\\ H_{ji}\left(\omega \right) & H_{jj}\left(\omega \right) \end{array}\right)\left(\begin{array}{c} \varepsilon \left(\omega \right)\\ \eta \left(\omega \right) \end{array}\right)+\left(\begin{array}{c} U\left(\omega \right)\\ U\left(\omega \right) \end{array}\right) \end{array}$$

Cast this way we see that the common signal *U*(ω) is exogenous to the system. We thus expect that the presence of a common signal will manifest as increased instantaneous interaction. However, whether the common signal will also distort the estimation of total Granger causality [which has been shown for the related case of measurement noise (Nalatore et al., 2007)] is less clear.

The same reasoning applies to the case of bipolar derivations that share a unipolar signal in their derivation. Substituting the expression for bipolar derivations [Equation (7) into (22)] we get

$$\begin{array}{l} \left(\begin{array}{c} X_{i}\left(\omega \right)\\ X_{i+1}\left(\omega \right) \end{array}\right)-\left(\begin{array}{c} X_{i+1}\left(\omega \right)\\ X_{i+2}\left(\omega \right) \end{array}\right)=\left(\begin{array}{cc} H_{ii}\left(\omega \right) & H_{ij}\left(\omega \right)\\ H_{ji}\left(\omega \right) & H_{jj}\left(\omega \right) \end{array}\right)\left(\begin{array}{c} \varepsilon \left(\omega \right)\\ \eta \left(\omega \right) \end{array}\right) \end{array}$$

By noting that spectral Granger causality is invariant under scalar multiplication (Geweke, 1982) we can use *bY*_*i*+1_(ω) → −*bY*_*i*+1_(ω) = *X*_*i*+2_(ω)−*X*_*i*+1_(ω). Rearranging in terms of the neural signals *X*_*i*_(ω) and *X*_*i*+2_(ω) we get

(24) $$\begin{array}{r} \left(\begin{array}{c} X_{i}\left(\omega \right)\\ X_{i+2}\left(\omega \right) \end{array}\right)=\left(\begin{array}{cc} H_{ii}\left(\omega \right) & H_{ij}\left(\omega \right)\\ H_{ji}\left(\omega \right) & H_{jj}\left(\omega \right) \end{array}\right)\left(\begin{array}{c} \varepsilon \left(\omega \right)\\ \eta \left(\omega \right) \end{array}\right)+\left(\begin{array}{c} X_{i+1}\left(\omega \right)\\ X_{i+1}\left(\omega \right) \end{array}\right) \end{array}$$

Equation (24) above has the same form as Equation (23). Thus, we expect that instantaneous interaction will be increased for bipolar derivations that share a unipolar signal in their derivation.

### Example simulations: a common signal increases instantaneous interaction

**Figure 4:**
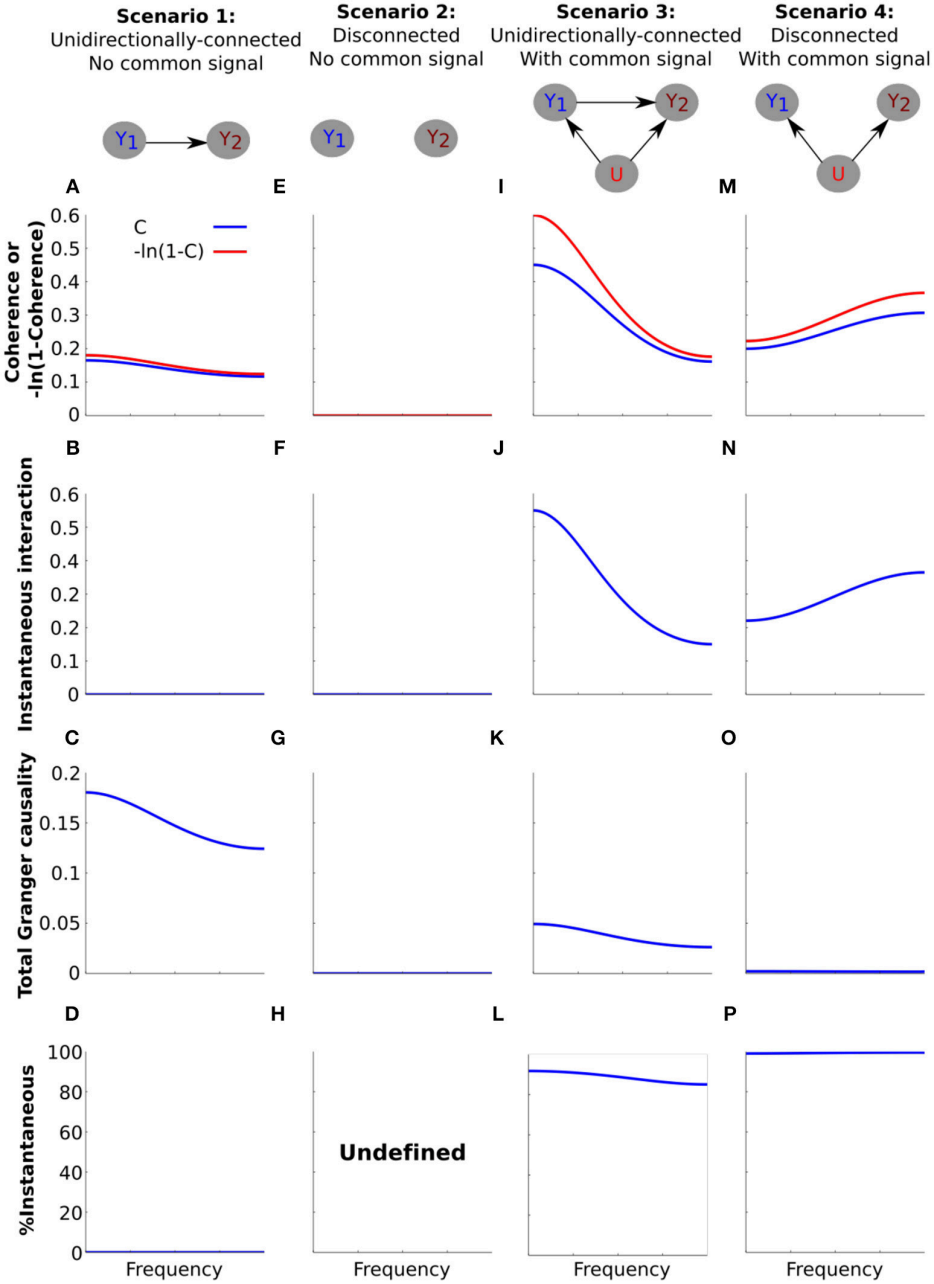
Simulating the effect of a common signal on instantaneous interaction and total Granger causality. Simulation results for **(A–D)** the unidirectionally-connected system without a common signal, **(E–H)** the disconnected system without a common signal, **(I–L)** the unidirectionally-connected system with a common signal, **(M–P**) the disconnected system with a common signal. **(A,E,I,M)** Transformed coherence (red) and coherence (blue) plotted on the same y-axis scale to facilitate comparison. (**B,F,J,N**) Instantaneous interaction. **(C,G,K,O)** Total Granger causality. **(D,H,L,P)** The percentage of transformed coherence that is accounted for by instantaneous interaction, computed as ($100*\frac{f_{1\cdot 2}(\omega )}{-\ln(1-C_{12})(\omega ))}$). For **(H)**, the percentage of transformed coherence is undefined due to the division by zero.

We next used simple simulations to illustrate how the decomposition in Equation (21) may manifest in empirical analysis. To do this we investigated the same four scenarios as for the coherence analysis in Figure 2; a unidirectionally-connected system and a disconnected system with no common signal, and the same two systems in the presence of an uncorrelated common signal (Figure 4). For each scenario we assessed the quantities in the decomposition in Equation (21); transformed coherence −ln (1−*C*(ω)), total Granger causality [*f*_1↔2_(ω)] and instantaneous interaction [*f*_1·2_(ω)].

For the unidirectionally-connected system (scenario 1) we have already seen that coherence values were low and decreased with frequency (Figure 2B). In order to decompose this system into total Granger causality and instantaneous interaction we first transformed coherence values into −ln (1−*C*(ω)). The transform is unlikely to affect interpretability of the results as it simply “stretches” coherence values. For example coherence values in the (0.01–0.99) range are stretched to the range (0.0101–4.6052). For the low values of coherence for scenario 1 the effect of the transform is minimal (Figure 4A).

Next we investigated instantaneous interaction. To do this we used the non-parametric Granger causality approach to decompose the spectral density matrix of the system (see Methods). Because the only dependency between the channels for the system is lagged, instantaneous interaction is zero (Figure 4B). Correspondingly, total Granger causality equals transformed coherence for this system (Figure 4C). Because our main interest is in the relationship between instantaneous interaction and coherence, we calculated the percentage of transformed coherence that is accounted for by instantaneous interaction ($100*\frac{f_{1\cdot 2}\left(\omega \right)}{-\ln\;\left(1-C_{12}\left(\omega \right)\right)}$) Figure 4D). Because instantaneous interaction is zero for this system, it accounts for none of the transformed coherence. For the disconnected system without a common signal (Figures 4E–G), coherence, instantaneous interaction and Granger causality are all zero (and % instantaneous interaction is undefined, Figure 4H).

We next investigated how the introduction of a common signal affects the estimation of instantaneous interaction and total Granger causality for the unidirectionally-connected system. We have seen that this results in an overall increase in coherences (Figures 2K,L). As we expected, the common signal substantially increased instantaneous interaction (Figure 4J, compared with Figure 4B). Furthermore, introduction of the common signal distorted the estimation of Granger causality; the total Granger causality was reduced (Figure 4K, compared with Figure 4C). Accordingly, the percentage of instantaneous interaction relative to the transformed coherence was very high, showing that most of the dependency between the channels is due to instantaneous interaction (Figure 4L).

Finally, we investigated how the common signal affects the estimation of instantaneous interaction and total Granger causality for the disconnected system (Figures 4M–P). The common signal increased coherence from 0 to 0.3 (Figure 4M, compared with Figure 4E), and magnitude increased with frequency (see also Figure 2L). Instantaneous interaction for this scenario was very high (Figure 4N) and, correspondingly, total Granger causality was very low (Figure 4O). As a result, transformed coherence for this system was almost entirely accounted for by instantaneous interaction (Figure 4P).

The key insight from these simple simulations is that the decomposition in Equation (21) can be applied to the analysis of real data. These simulations also demonstrate the different ways in which a common signal can affect total Granger causality and coherence. For example, we saw that a common signal results in high coherence even for a disconnected system (Figures 2L, 4M). In contrast, total Granger causality for the same scenario remained near zero (Figure 4O). For the unidirectionally-connected system the common signal increased coherence (Figures 2K, 4I) but decreased total Granger causality (Figure 4K). Thus, the effects of a common signal on Granger causality do not directly follow from the effects of the common signal on coherence. However, once instantaneous interaction, so far neglected in empirical analysis, is also taken into account, the decomposition in Equation (21) is completed and a much clearer picture emerges.

### Granger causality: results from analysis of fly LFP

#### Instantaneous interaction accounts for the high coherence observed for the unipolar signals

**Figure 5:**
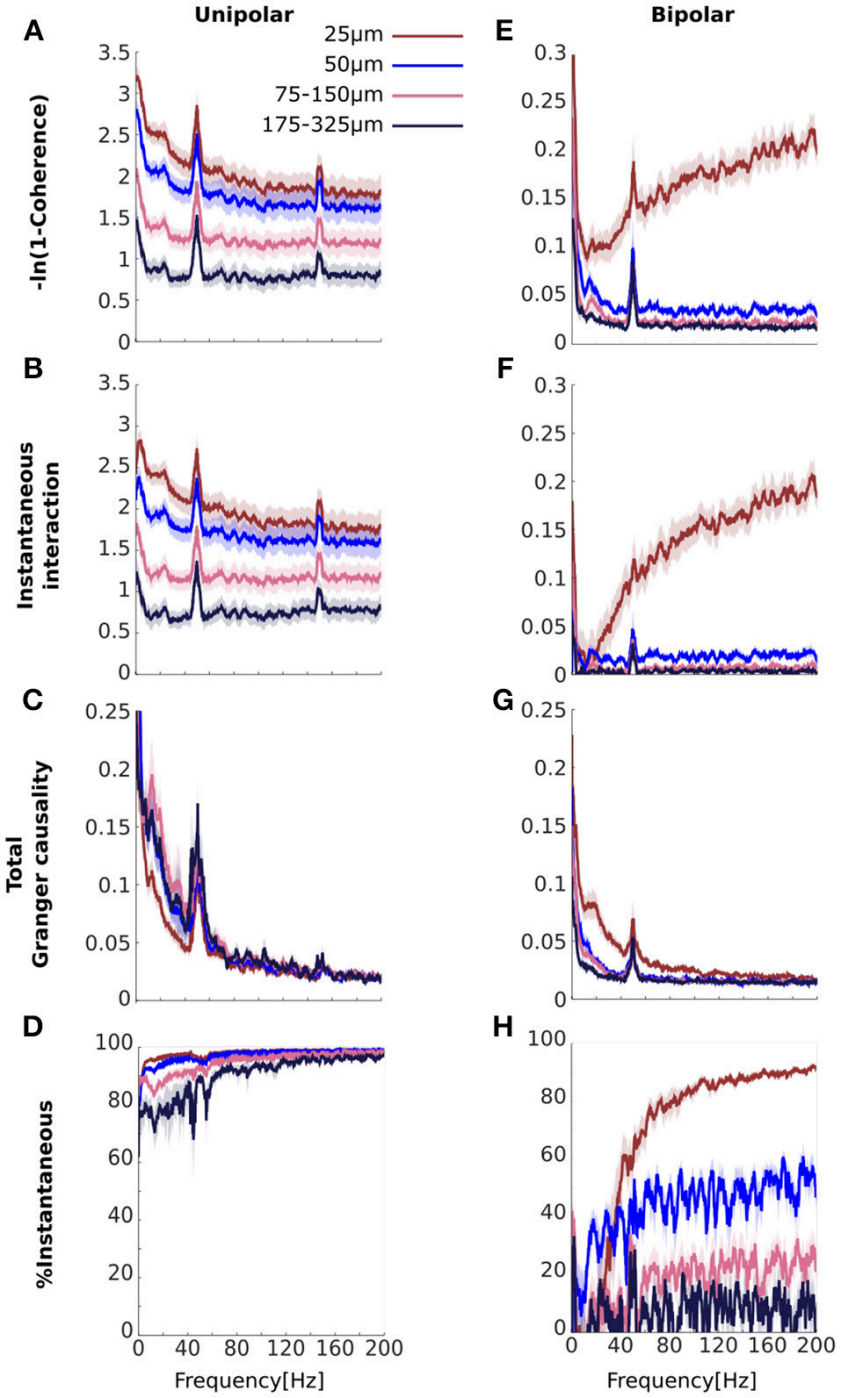
Comparisons of the results for transformed coherence, instantaneous interaction and total Granger causality computed from the experimental data **(A–D)** for unipolar signals and **(E–H)** for bipolar derivations. **(A, E)** Transformed coherence **(B,F)**, instantaneous interaction, **(C,G)**, total Granger causality, and **(D,H)** the percentage of transformed coherence that is accounted for by instantaneous interaction. The color of each line represents the separation between signal pairs (brown for 25 μm, blue for 50 μm, pink for 75–150 μm and black for 175–325 μm, see Methods for details). Bipolar derivations separated by 25 μm share a unipolar signal in their derivation. Peaks at 50 Hz and 150 Hz are due to line noise. Shaded area represents sem across flies (*N* = 13).

Our simulations demonstrate that a common signal may manifest as increased instantaneous interaction. If the high coherences we observed for unipolar signals (Figure 3A) are a result of common signals then we would expect that these would also manifest as increased instantaneous interaction. To investigate this, we decomposed (transformed) coherence into total Granger causality and instantaneous interaction (Figures 5A–D). Due to the transformation, the original unipolar coherence, ranging from ~0.5 to 0.9 (Figure 3A), were re-scaled to ~0.7 to 3.0 (Figure 5A). Similar to coherence, transformed coherence decreased with increasing distance between the electrodes but remained high even for unipolar signals that were far apart (175–325 μm). Transformed coherence closely resembled instantaneous interaction in all respects (Figure 5B). Correspondingly, we found that total Granger causality, which captures only lagged effects, was relatively small (Figure 5C). Further, total Granger causality values approached zero as frequency increased, as we would expect if there was no genuine neurophysiological coupling at those frequencies. In sum, this suggests that most of the unipolar coherence was due to instantaneous interaction, which we confirmed by calculating the percentage of transformed coherence that is accounted for by instantaneous interaction (Figure 5D). Together, these findings strongly suggest the presence of common signals and that these manifest as increased instantaneous interaction.

#### Instantaneous interaction accounts for the increasing coherence between bipolar derivations that share a unipolar signal in their derivation

We have shown that the high unipolar coherences were accounted for by instantaneous interaction, strongly indicating the presence of common signals. Bipolar coherences were generally lower (Figure 3B), which indicates that bipolar derivations were largely free from common signals. However, coherence for bipolar derivations that shared a unipolar signal in their derivation increased with frequency. We attribute this increase to the unipolar signal shared in their derivation (Figure 3B). If this signal is the source of the increasing coherence with frequency, then we would expect this increase in coherence to be accounted for by instantaneous interaction.

We repeated the analysis for the bipolar derivations (Figures 5E–H). For the bipolar derivations, transformed coherence was very similar to the original coherence, including the important characteristic that transformed coherence increased with frequency for bipolar derivations that shared a unipolar signal in their derivation (25 μm separation shown in brown, Figure 5E). Instantaneous interaction (Figure 5F) was much smaller than that for the unipolar signals (Figure 5B), indicating that bipolar derivations were largely free from common signals. We also found that instantaneous interaction increased with frequency for bipolar derivations that share a unipolar signal in their derivation, but not for pairs derived from independent unipolar signals. This observation is in line with our prediction that this increase was due to the unipolar signal shared in their derivations. Consistent with this, total Granger causality for these pairs did not increase with frequency, indicating that the increase cannot be attributed to any lagged influences (Figure 5G). Indeed, total Granger causality appeared to decrease with increasing frequency for all bipolar pairs. Figure 5H summarizes these results as the percentage of transformed coherence that is accounted for by instantaneous interaction. This clearly showed that the increasing coherence with frequency for bipolar derivations that share a unipolar signal is dominated by instantaneous interaction. However, even for bipolar pairs separated by 50 μm, which were derived from independent unipolar signals (blue in Figure 5H), instantaneous interaction substantially (~40–60%) contributed to transformed coherence. This could indicate that bipolar derivations are not *completely* free of common signals.

We remind the reader that our Granger causality analysis is based on autoregressive process. Autoregressive-based application of Granger causality is the most common but does involve assumptions regarding stationarity and linearity. It is possible that causality estimation in our data using improved methods that do not require such assumptions (e.g., Sheikhattar et al., 2018) would be differently affected by common signals and bipolar referencing.

## Discussion

In this paper, we investigated the effects of common signals on power, coherence and Granger causality. We did this through a combination of theoretical review, simulations and empirical analysis of fly LFPs. The theoretical background sections aggregated known results in a simple way and using unified terminology. The important theoretical concepts were complemented by illustrative simulation using the autoregressive framework on which Granger causality is based. Basing the simulations on autoregressive processes also allowed the estimation of power and coherence directly from the autoregressive parameters, and is in line with previous Granger causality (Ding et al., 2006; Dhamala et al., 2008; Nalatore et al., 2009; Chicharro, 2012; Wen et al., 2013) and coherence work (Rappelsberger, 1989). Codes for the simulations are publicly available from https://github.com/DrorGitHub/A-unified-framework-for-dissecting___simulations.

Our empirical analysis of LFPs recorded from the brains of flies resulted in a number of novel findings. First, we showed that substantial common signals were indeed present in the linear array electrodes used for our experiments. The common signals are likely to reflect electrical activity at the reference electrode as well as volume conduction from other sources of electrical activity. Second, we showed that bipolar derivations largely removed the effects of these common signals. Third, through theoretical analysis and simulation, we showed that in special cases bipolar derivations can actually introduce another common signal, due to a shared unipolar signal. These findings establish that simple and well known techniques such as bipolar derivations commonly used in the analysis of human EEG and mammalian LFPs studies are also suitable for the analysis of LFPs recorded using very closely spaced electrode arrays, and that similar pitfalls should also be observed. We also showed that instantaneous interaction increased in the presence of common signals. We suggest therefore that instantaneous interactions can serve as a useful indicator for checking for common signals in other recording preparations.

Based on our review of theory, simulations and results from the analysis of fly LFPs we suggest the following for checking for the presence of common signals. First, substantially greater unipolar than bipolar power is an indication of the presence of common signals. Second, substantially greater unipolar than bipolar coherence is an indication of the presence of common signals. Third, substantially greater unipolar instantaneous interaction than bipolar instantaneous interaction may indicate the presence of common signals.

In particular, high coherence and/or instantaneous interaction in high frequencies for which we would not expect genuine neurophysiological coupling indicates the presence of common signals. We observed all these criteria in our data, strongly indicating the presence of common signals.

In our data unipolar coherence was high, even for high frequencies for which we would not expect genuine neurophysiological coupling (Figure 3A). Coherence in high frequencies was highest for pairs separated by 25 μm, but remained well above zero even for pairs separated by 175–325 μm. This demonstrates that common signals affect even electrode pairs that are comparatively far apart, as would be expected from a common signal due to electrical activity at the reference. The reduction in unipolar coherence in high frequencies with increasing separation between the signals is consistent with common signals due to volume conduction from other electrical sources. In the future, by modeling how neural signals propagate through the brain (e.g., Bedard et al., 2004; Rudolph and Destexhe, 2006; Gomes et al., 2016; Miceli et al., 2017), we may be able to distinguish between the common signals from electrical activity at the reference and those from volume conduction of other electrical sources. Sub-network analysis may also be adapted for this purpose (Elsegai et al., 2015).

For the bipolar derivations, however, coherence in high frequencies for electrode pairs, separated by 50 μm to those separated by 175–325 μm was near zero (Figure 3B), indicating that bipolar derivations largely removed the effects of the common signals, irrespective of whether these were due to electrical activity at the reference or due to volume conduction from other electrical sources (see Appendix A in Supplementary Materials for detail). Similar observations held for instantaneous interaction (i.e., highest for pairs separated by 25 μm, but remained well above zero even for pairs separated by 175–325 μm, and substantially reduced for the bipolar derivations, Figures 5B–F).

Aside from our empirical findings, one unique aspect of our paper is the combined treatment of power, coherence and Granger causality. While it is straightforward to assess the effect of common signals on these quantities in isolation, empirical studies often analyze all three quantities (Brovelli et al., 2004; Barrett et al., 2012; Bastos et al., 2014; Fontolan et al., 2014; Van Kerkoerle et al., 2014; Michalareas et al., 2016). This is one reason we investigated all three quantities together. The progression from the analysis of power to coherence to Granger causality is natural in that it follows the progression in complexity of these quantities. From a theoretical perspective, our review demonstrates how the power of the common signals affects the coherence estimate (Equation 12). Further, Equation (40) shows how the power of the common signals affects the cross spectral density matrix, and in turn the Granger causality estimates. Finally, coherence and Granger causality are analytically related (Equation 21). Indeed, our proposal to investigate instantaneous interaction follows from the analytical relationship between these quantities. Thus, we think that the combined analysis of power, coherence, and Granger causality provides a more holistic picture of the effects of the common signals.

We believe that our holistic treatment of the effects of common signals on power, coherence and Granger causality using theory, simulation and empirical analysis serves as a solid foundation for analyzing empirical neural data. However, the suitability of the framework's assumptions for analyzing empirical data requires care. First, the framework rests on the assumption that the recorded activity can be represented as the sum of ongoing neural activity and common signals. As such, the framework is suited for guiding the analysis of spontaneous, not evoked, activity, as it does not currently consider the presence of a stimulus. If the only data available corresponds to evoked activity then “spontaneous” activity may be estimated by removing the evoked component (by, for example, averaging across repeated presentations). In such practice, however, care is required to ensure that the resulting data may be reasonably treated as “spontaneous” (Truccolo et al., 2002; Wang et al., 2008).

Second, in our mathematical treatment we have assumed that the common signals are independent of the neural activity, as this results in relatively simple expressions and is also in line with previous work (e.g., Rappelsberger, 1989; Nunez et al., 1997; Essl and Rappelsberger, 1998; Hu et al., 2010). In our simulations (Figures 2, 4) we were able to satisfy this assumption by construction. However, this may not always be reflective of real data. For example if the reference electrode is located inside the brain then the activity at the reference electrode is unlikely to be independent of the neural activity of interest. Even in such a case, the full expressions for power and coherence still hold [e.g., Equations (3), (10), and (17)], but inference about any common signals will become more difficult. (See Strube-Bloss et al., 2012 for an alternative approach using PCA).

Third, we have assumed that instantaneous interaction is physically meaningful. This assumption requires special care because a formal link between instantaneous interaction and common signals has not been demonstrated. However, we note that some results are available for the related case of measurement noise (Nalatore et al., 2007). In addition, concerns about the interpretability of instantaneous interaction have been expressed because it can become negative in certain situations (Ding et al., 2006; Chicharro, 2012; Wen et al., 2013). We acknowledge this concern, but also point out that our work here suggests that this quantity may be at least of *empirical* value. For example, it can be used as a diagnostic for the presence of common signals, as we showed here. It would be interesting to compare instantaneous interaction with other methods used to assess instantaneous influences in the context of Granger causality (Faes et al., 2013; Vinck et al., 2015).

We also clarify that even though Granger causality quantifies lagged effects, its estimation in the presence of common signals maybe misleading (Nalatore et al., 2007; Friston et al., 2014; Bastos and Schoffelen, 2016; Trongnetrpunya et al., 2016). Indeed, in our simulations the presence of a common signal resulted in lower Granger causality values (Figure 4K) than the ground truth without a common signal (Figure 4C). With our analysis of the unipolar data (which contained substantial common signals), total Granger causality values in lower frequencies (<50 Hz) for adjacent pairs (separated by 25 μm) were actually smaller than those for pairs that were separated by more than 50 μm, which is physiologically highly questionable (Figure 5C). With our analysis of the bipolar data (which contained little common signals), however, higher total Granger causality for closer pairs were observed in low frequency range (<50 Hz, Figure 5G), which is much more physiologically plausible.

## Conclusions and future work

In this work we investigated the effects of common signals through theory, simulation and empirical analysis of local field potentials recorded using linear electrodes from the fly brain. In particular, we investigated in detail the effects of common signals on unipolar signals and bipolar derivations.

We did not consider numerous other techniques for reducing common signals, such as other referencing techniques (e.g., Rappelsberger, 1989; Nunez et al., 1997; Essl and Rappelsberger, 1998), methods based on Independent or Principal Component analysis (e.g., Yao et al., 2005; Hu et al., 2010; Madhu et al., 2012) or other linear decompositions (Brookes et al., 2012; Drakesmith et al., 2013; Colclough et al., 2015), or methods based on more detailed modeling of the dynamics (e.g., Nalatore et al., 2007, 2009; Faes et al., 2013; Friston et al., 2014). We also did not consider multivariate approaches, such as partial coherence (Kocsis et al., 1999; Bendat and Piersol, 2000) and partial or conditional Granger causality (Guo et al., 2008; Wen et al., 2013; Barnett and Seth, 2014). These methods can all be used to address the adverse effects of common signals and the possibility of dissecting inputs from other neural sources. However, they also introduce their own complications. For example, partialization on a third signal requires that that signal is known or recorded. In the case of the effect of another neural region, that may be often the case. However, we typically do not have access to the electrical activity at the reference electrode, and so we cannot directly partialize it out. Another drawback of partialization is that it requires estimating more parameters, making it inherently more complex compared to pairwise analysis. In general, as the number of the variables to estimate increases, the more data is required to achieve the same level of estimation accuracy. There is also no guarantee that partialization will be complete in its removal of common signals. Compared to this, bipolar derivations + pairwise analysis are far simpler and more widely adopted, making them a reasonable choice for investigation in the manuscript (Bastos et al., 2014; Bastos and Schoffelen, 2016; Trongnetrpunya et al., 2016). Nonetheless, we view the investigation of multivariate techniques as a potentially important extension of our work.

We did not assess the many other functional and effective connectivity techniques (e.g., Greenblatt et al., 2012; Wang et al., 2014; Bastos et al., 2015). Thus, we do not claim that our findings generalize to these other techniques. For example, the Granger causality analysis we carried out is based on autoregressive process. Autoregressive-based application of Granger causality is the most widespread but involves comparatively restrictive assumptions regarding stationarity and linearity. Recent improvements in causal analysis methods can handle non-stationarity and non-linearity and are potentially better suited for the analysis of empirical data (e.g., Sheikhattar et al., 2018). It is possible that causality estimation in our data using these more advanced methods would be differently affected by common signals and bipolar referencing.

We focused on bipolar derivations together with coherence and autoregressive-based Granger causality metrics as these are widely adopted (e.g., Chen et al., 2006; Ding et al., 2006; Dhamala et al., 2008; Bressler and Seth, 2010; Blinowska, 2011; Cimenser et al., 2011; Barnett and Seth, 2014; Buzsáki and Schomburg, 2015; Seth et al., 2015; Bastos and Schoffelen, 2016; Bowyer, 2016). In addition coherence and Granger causality can be analytically related in a (relatively) straightforward manner. By exploiting analytic relationships between other connectivity metrics it may be possible to gain an even deeper understanding into the nature of any common signals. Our approach combined with other methods for assessing common signals (Shahbazi et al., 2010; Haufe et al., 2013; Elsegai et al., 2015; Winkler et al., 2016) will reduce the chance of misinterpreting functional and effective connectivity analysis, the key analysis techniques in modern systems and cognitive neuroscience (Gregoriou et al., 2009; Boveroux et al., 2010; Bressler and Seth, 2010; Supp et al., 2011; Bosman et al., 2012; Horwitz and Horovitz, 2012; Hudetz and Mashour, 2016; Rosenberg et al., 2016).

### Methods

In the main text, we have provided the core theoretical background, simulation, and analysis of the experimental data. Here, we provide further methodological details. We first cover the mathematical formulation of spectral Granger causality. We then provide full details of the simulations, followed by complete details of the LFPs data analysis.

#### Spectral Granger causality

In this section we briefly recap the theoretical background for spectral Granger causality. Here we focus on the lesser-known relationship between coherence, Granger causality and instantaneous interaction and we only rehearse the relevant components to our analysis, generally following the treatment of Ding et al. (2006), and Wen et al. (2013). More complete details can be found in Geweke (1982, 1984), Lütkepohl (2005), Dhamala et al. (2008), Chicharro (2012), Wen et al. (2013), Wibral et al. (2014), and Seth et al. (2015).

In simple terms, a signal *y*_*i*_ is said to *Granger-cause* a signal *y*_*j*_ if past values of *y*_*i*_ improve predictions of future values of *y*_*j*_. This notion is quantified using the framework of autoregressive processes.

Consider two stationary time series represented by the standard autoregressive process

(25) $$\begin{array}{l} y_{i}(t)=-\sum_{l=1}^{\infty }a(l)y_{i}(t-l)-\sum_{l=1}^{\infty }b(l)y_{j}(t-l)+\varepsilon (t)\\ y_{j}(t)=-\sum_{l=1}^{\infty }c(l)y_{i}(t-l)-\sum_{l=1}^{\infty }d(l)y_{j}(t-l)+\eta (t) \end{array}$$

where *a, b, c*, and *d* represent the autoregressive coefficients and the index *l* represents the lag. ε(*t*) and η(*t*) are zero-mean Gaussian noise sources with covariance matrix given by

$$\begin{array}{l} \Sigma =\left(\begin{array}{cc} \Sigma & \gamma \\ \gamma & \Gamma \end{array}\right) \end{array}$$

where var(ε(*t*)) = Σ, var(η(*t*)) = Γ and cov(ε(*t*), η(*t*)) = γ.

To obtain the spectral formulation of Granger causality we first express the autoregressive process in the frequency domain. By introducing the polynomial lag operator,

$$\begin{array}{l} \varphi \left(L\right)=\overset{\infty }{\underset{l=0}{\sum }}\varphi \left(l\right)L^{l} \end{array}$$

where the operator *L*^*l*^ acts on a function y_i_(t) as, $L^{l}y_{i}\left(t\right)=y_{i}\left(t-l\right)$. With this notation, we can rewrite Equation (25) in matrix form as

(26) $$\begin{array}{r} \left(\begin{array}{cc} a\left(L\right) & b\left(L\right)\\ c\left(L\right) & d\left(L\right) \end{array}\right)\left(\begin{array}{c} y_{i}\left(t\right)\\ y_{j}\left(t\right) \end{array}\right)=\left(\begin{array}{c} \varepsilon \left(t\right)\\ \eta \left(t\right) \end{array}\right) \end{array}$$

where *a*(0) = 1, *b*(0) = 0, *c*(0) = 0, *d*(0) = 1. Taking the Fourier transform of both sides gives

(27) $$\begin{array}{r} \left(\begin{array}{cc} a\left(\omega \right) & b\left(\omega \right)\\ c\left(\omega \right) & d\left(\omega \right) \end{array}\right)\left(\begin{array}{c} Y_{i}\left(\omega \right)\\ Y_{j}\left(\omega \right) \end{array}\right)=\left(\begin{array}{c} \varepsilon \left(\omega \right)\\ \eta \left(\omega \right) \end{array}\right) \end{array}$$

By using $\left(\begin{array}{cc} H_{ii}\left(\omega \right) & H_{ij}\left(\omega \right)\\ H_{ji}\left(\omega \right) & H_{jj}\left(\omega \right) \end{array}\right)={\left(\begin{array}{cc} a\left(\omega \right) & b\left(\omega \right)\\ c\left(\omega \right) & d\left(\omega \right) \end{array}\right)}^{-1}$we can rewrite Equation (27) in transfer function format as

(28) $$\begin{array}{r} \left(\begin{array}{c} Y_{i}\left(\omega \right)\\ Y_{j}\left(\omega \right) \end{array}\right)=\left(\begin{array}{cc} H_{ii}\left(\omega \right) & H_{ij}\left(\omega \right)\\ H_{ji}\left(\omega \right) & H_{jj}\left(\omega \right) \end{array}\right)\left(\begin{array}{c} \varepsilon \left(\omega \right)\\ \eta \left(\omega \right) \end{array}\right) \end{array}$$

The spectral density matrix is obtained by multiplying both sides by the conjugate transpose of each side, denoted by ^*^, yielding

(29) $$\begin{array}{r} \left(\begin{array}{cc} S_{ii}\left(\omega \right) & S_{ji}\left(\omega \right)\\ S_{ij}\left(\omega \right) & S_{jj}\left(\omega \right) \end{array}\right)=\left(\begin{array}{cc} H_{ii}\left(\omega \right) & H_{ij}\left(\omega \right)\\ H_{ji}\left(\omega \right) & H_{jj}\left(\omega \right) \end{array}\right)\left(\begin{array}{cc} \Sigma & \gamma \\ \gamma & \Gamma \end{array}\right)\\ {\left(\begin{array}{cc} H_{ii}\left(\omega \right) & H_{ij}\left(\omega \right)\\ H_{ji}\left(\omega \right) & H_{jj}\left(\omega \right) \end{array}\right)}^{*} \end{array}$$

This can be compactly written in matrix form as

(30) $$\begin{array}{r} \text{Q}\left(\omega \right)=\text{H}\left(\omega \right)\Sigma {\text{H}}^{*}\left(\omega \right) \end{array}$$

where $H(\omega )=\left(\begin{array}{cc} H_{ii}(\omega ) & H_{ij}(\omega )\\ H_{ji}(\omega ) & H_{jj}(\omega ) \end{array}\right)$ and $Q(\omega )=\left(\begin{array}{cc} S_{ii}(\omega ) & S_{ji}(\omega )\\ S_{ij}(\omega ) & S_{jj}(\omega ) \end{array}\right)$.

The diagonal elements of the spectral density matrix **Q**(ω) correspond to the auto-spectra and the off diagonal elements correspond to the cross-spectra (Wen et al., 2013). Note that coherence can be directly calculated from the spectral density matrix (Equation 9).

Spectral measures of total interdependence *f*_*i,j*_(ω), GC influence from Yi to Yj *f*_*i*→*j*_(ω) and GC influence from Yj to Yi *f*_*i*←*j*_(ω) are defined as

(31) $$\begin{array}{r} f_{i,j}\left(\omega \right)=\ln\;\frac{S_{ii}\left(\omega \right)S_{jj}\left(\omega \right)}{\left|\text{Q}\left(\omega \right)\right|} \end{array}$$

(32) $$\begin{array}{r} f_{i\to j}\left(\omega \right)=\ln\;\frac{S_{jj}\left(\omega \right)}{{\tilde{H}}_{jj}\left(\omega \right)\Gamma {\tilde{H}}_{jj}^{*}\left(\omega \right)} \end{array}$$

(33) $$\begin{array}{r} f_{i\leftarrow j}\left(\omega \right)=\ln\;\frac{S_{ii}\left(\omega \right)}{{\tilde{H}}_{ii}\left(\omega \right)\Sigma {\tilde{H}}_{ii}^{*}\left(\omega \right)} \end{array}$$

where ${\tilde{H}}_{jj}\left(\omega \right)=H_{jj}\left(\omega \right)+\left(\gamma /\Gamma \right)H_{ij}\left(\omega \right)$ and ${\tilde{H}}_{ii}\left(\omega \right)=H_{ii}\left(\omega \right)+\left(\gamma /\Sigma \right)H_{ji}\left(\omega \right)$. These transformations are irrelevant if γ = 0.

A simple transform relates coherence and total interdependence

(34) $$\begin{array}{r} f_{i,j}\left(\omega \right)=\ln\;\frac{S_{ii}\left(\omega \right)S_{jj}\left(\omega \right)}{\left|\text{Q}\left(\omega \right)\right|}\\ =\ln\;\left(\frac{S_{ii}\left(\omega \right)S_{jj}\left(\omega \right)}{S_{ii}\left(\omega \right)S_{jj}\left(\omega \right)-S_{ij}\left(\omega \right)S_{ij}^{*}\left(\omega \right)}\right)\\ =-\ln\;\left(\frac{S_{ii}\left(\omega \right)S_{jj}\left(\omega \right)-S_{ij}\left(\omega \right)S_{ij}^{*}\left(\omega \right)}{S_{ii}\left(\omega \right)S_{jj}\left(\omega \right)}\right)\\ =-\ln\;\left(1-\frac{S_{ij}\left(\omega \right)S_{ij}^{*}\left(\omega \right)}{S_{ii}\left(\omega \right)S_{jj}\left(\omega \right)}\right)\\ =-\ln\;\left(1-\frac{abs{\left(S_{ij}\left(\omega \right)\right)}^{2}}{S_{ii}\left(\omega \right)S_{jj}\left(\omega \right)}\right)\\ =-\ln\;\left(1-C_{ij}\left(\omega \right)\right) \end{array}$$

By subtracting *f*_*i*→*j*_(ω) and *f*_*i*←*j*_(ω) from *f*_*i,j*_(ω) we can get what is known as “instantaneous causality” [*f*_*i*▪*j*_(ω) (Ding et al., 2006; Wen et al., 2013)]

(35) $$\begin{array}{r} f_{i\cdot j}\left(\omega \right)=f_{i,j}\left(\omega \right)-f_{i\to j}\left(\omega \right)-f_{i\leftarrow j}\left(\omega \right)\\ =\ln\;\frac{\left({\tilde{H}}_{ii}\left(\omega \right)\Sigma {\tilde{H}}_{ii}^{*}\left(\omega \right)\right)\left({\tilde{H}}_{jj}\left(\omega \right)\Gamma {\tilde{H}}_{jj}^{*}\left(\omega \right)\right)}{\left|\text{Q}\left(\omega \right)\right|} \end{array}$$

However, we feel that the term *instantaneous causality* is confusing in so far as (Granger) *causality* reflects *time-lagged* influences. For this reason we refer to this term as *instantaneous interaction* throughout the manuscript.

Using instantaneous interaction and the Granger causal influences we obtain the following decomposition of total interdependence

(36) $$\begin{array}{r} f_{i,j}\left(\omega \right)=f_{i\cdot j}\left(\omega \right)+f_{i\to j}\left(\omega \right)+f_{i\leftarrow j}\left(\omega \right) \end{array}$$

The relationship between coherence, Granger causality and instantaneous interaction is given by

(37) $$\begin{array}{r} -\ln\;\left(1-C_{ij}\left(\omega \right)\right)=f_{i,j}\left(\omega \right)=f_{i\cdot j}\left(\omega \right)+f_{i\to j}\left(\omega \right)+f_{i\leftarrow j}\left(\omega \right) \end{array}$$

If we disregard the directionality of the influence, we can simply contrast “lagged” (*f*_*i*→*j*_(ω) + *f*_*i*←*j*_(ω)) and “instantaneous” (*f*_*i*·*j*_(ω)) influences. To do this we introduce *total Granger causality f*_*i*↔*j*_(ω)

(38) $$\begin{array}{r} f_{i↔j}\left(\omega \right)=f_{i\to j}\left(\omega \right)+f_{i\leftarrow j}\left(\omega \right) \end{array}$$

We thus rewrite Equation (37) as

(39) $$\begin{array}{r} -\ln\;\left(1-C_{ij}\left(\omega \right)\right)=f_{i↔j}\left(\omega \right)+f_{i\cdot j}\left(\omega \right) \end{array}$$

This equation demonstrates that under the autoregressive framework of Granger causality, (a transformation of) coherence [left side of (39)] can be decomposed into total Granger causality [first term on the right side (39)], which capture lagged influences, and instantaneous interaction [second term on the right side of (39)], which captures any remaining instantaneous influences, possibly due to exogenous sources. This relationship forms the basis of our simulation examples and experimental data analysis.

#### Non-parametric estimation of spectral Granger causality

Spectral Granger causality analysis can be carried out either parametrically or non-parametrically. In the parametric approach the autoregressive model in Equation (25) is first fit to the data. Once the parameters of the models have been estimated Equations (26)–(30) are used to obtain the transfer function **H**(ω), noise covariance matrix Σ and spectral density matrix **Q**(ω). Using these quantities *f*_*i*→*j*_(ω), *f*_*i*←*j*_(ω) and *f*_*i*·*j*_(ω) can be calculated as per Equations (32), (33), and (35).

In the non-parametric approach proposed in Dhamala et al. (2008), **H**(ω) and Σ are obtained directly by factorizing the spectral density matrix **Q**(ω). This corresponds to obtaining the right side of Equation (30) directly from its left using a factorization procedure described by Wilson (1972), without explicitly fitting the autoregressive model. Thus, non-parametric estimation is entirely dependent on the estimation of the spectral density matrix. One advantage of non-parametric over parametric estimation of GC influences is that it does not require specification of the autoregressive model order (Dhamala et al., 2008).

Recall that coherence (Equation 9) is also directly estimated from the spectral density matrix. Thus, once the spectral density matrix has been estimated, power, coherence, total Granger causality, and instantaneous interaction can all be derived. For our simulations the spectral density matrix is obtained analytically while for the experimental sections the spectral density matrix is estimated empirically. We provide further details on these below.

#### Simulations

In our simulations we explored how coherence, total Granger causality and instantaneous interaction are affected by the presence of a common signal. For our simulations we used the autoregressive framework. The autoregressive dynamics we simulated are likely much simpler than the recorded fly LFPs. However, Granger causality as assessed here is *defined* for autoregressive processes (see above). Investigating other types of dynamics would violate the Granger causality assumptions from the outset, making interpretation difficult and defeating the purpose of the simulations. In addition, power and coherence can also be directly estimated from the autoregressive parameters, obviating the need for any numerical methods. Our use of the autoregressive framework is also consistent with previous Granger causality (Ding et al., 2006; Dhamala et al., 2008; Nalatore et al., 2009; Chicharro, 2012; Wen et al., 2013) and coherence work (Rappelsberger, 1989).

The simulations were not intended for systematically testing the effects of *all* types of common signals, which may depend on specific recording setups and the biophysical characteristics of the recording preparations. Instead, we designed the simulations to highlight the theoretical concepts and to serve as a simplified precursor to the empirical analysis. To do this we explored four scenarios. The first two scenarios correspond to a unidirectionally-connected and a disconnected (meaning that the cross-spectrum between the signals is zero for all frequencies) system. These two scenarios establish the “ground truth” for coherence, total Granger causality and instantaneous interaction in the absence of a common signal. The remaining two scenarios examine how these quantities are affected by a common signal. Because disconnected and unidirectionally-connected models were sufficiently complex to model non-trivial behavior, we opted not to present more complex scenarios, such as those involving bidirectionally-connected models.

Following the notation of the unipolar signals the four scenarios can be described as

**Table:** 

	Unidirectionally-connected ($X_{1}\left(\omega \right)X_{2}^{*}\left(\omega \right)\neq 0$)	Disconnected (${X^{\prime }}_{1}\left(\omega \right){X^{\prime }}_{2}^{*}\left(\omega \right)=0$)
No common signal	Scenario 1 *y*_1_(*t*) = *x*_1_(*t*)	Scenario 2 $y_{1}^{\prime }\left(t\right)=x_{1}^{\prime }\left(t\right)$
	*y*_2_(*t*) = *x*_2_(*t*)	$y_{2}^{\prime }\left(t\right)=x_{2}^{\prime }\left(t\right)$
With common signal	Scenario 3 *y*_1_(*t*) = *x*_1_(*t*)+*u*(*t*)	Scenario 4 $y_{1}^{\prime }\left(t\right)=x_{1}^{\prime }\left(t\right)+u\left(t\right)$
	*y*_2_(*t*) = *x*_2_(*t*)+*u*(*t*)	$y_{2}^{\prime }\left(t\right)=x_{2}^{\prime }\left(t\right)+u\left(t\right)$

We clarify that these scenarios are only indirectly related to the choice of referencing scheme. Instead, these simulations directly investigate the effects of a common signal. This can correspond to either unipolar signals which are affected by a common signal from the reference electrode, or to bipolar derivations which share a unipolar signals in their derivation.

#### Simulation framework

For our simulations we used *a* = 0.1, *c* = 0.4, and *d* = 0.1 in Equation (20). These parameter settings result in power and coherence spectra that are roughly reflective of biological systems (Figures 2E, I). The noise terms were chosen as uncorrelated noise sources with unit standard deviation.

When modeling the disconnected system we ensured that the power spectra equaled the power spectra of the unidirectionally-connected system. That is, we set

$$\begin{array}{l} X_{1}(\omega )X_{1}^{*}(\omega )={X^{\prime }}_{1}(\omega ){X^{\prime }}_{1}^{*}(\omega )\\ X_{2}(\omega )X_{2}^{*}(\omega )={X^{\prime }}_{2}^{(}\omega ){X^{\prime }}_{2}^{*}(\omega )\\ {X^{\prime }}_{1}(\omega ){X^{\prime }}_{2}^{*}(\omega )=0 \end{array}$$

We aimed to do this because coherence, total Granger causality and instantaneous interaction depend on the power spectra of the signals [see Equation (9) for coherence and Equation (32) for Granger causality]. By equating the power we ensured that the two systems are not distinguishable based on the power alone. Note that simply setting the autoregressive coefficient *d* = 0 in Equation (20) would ensure the system is disconnected but would also change the power spectrum of *X*_1_(ω).

We modeled the common signal for scenarios 3 and 4 as an uncorrelated white noise signal, u(t). We set the power of the signal [*U*(ω)U^*^(ω)] to the mean power of the neural signals across frequencies

$$U\left(\omega \right)U^{*}\left(\omega \right)=\frac{1}{2\omega_{NQ}}\overset{\omega =NQ}{\underset{\omega =0}{\sum }}\left(X_{1}\left(\omega \right)X_{1}^{*}\left(\omega \right)+X_{2}\left(\omega \right)X_{2}^{*}\left(\omega \right)\right)$$

where ω_*NQ*_ corresponds to the Nyquist frequency which is arbitrary for our simulation. Matlab code for the simulations is available from https://github.com/DrorGitHub/A-unified-framework-for-dissecting___simulations.

#### Simulations for the unidirectionally-connected system (scenarios 1 and 3)

As mentioned above, the key quantity to estimate is the spectral density matrix, from which power, coherence, total Granger causality and instantaneous interaction can all be derived.

For the unidirectionally-connected system with no common signal (scenario 1) one can use the autoregressive description of the system and Equations (26)–(30) to obtain the spectral density matrix $Q(\omega )=\left(\begin{array}{cc} S_{11}(\omega ) & S_{12}(\omega )\\ S_{21}(\omega ) & S_{22}(\omega ) \end{array}\right)$. The spectral density matrix of an autoregressive process can be obtained using the MATLAB (MathWorks) function *ft_freqanalysis*.m function from the FieldTrip toolbox (Oostenveld et al., 2010). Coherence and instantaneous interaction are obtained using the FieldTrip function *ft_connectivityanalysis.m*. Total Granger causality is obtained by first assessing the Granger causal influences from y1 to y2 and from y2 and y1 (also using *ft_connectivityanalysis.m*) and summing them as per Equation (38).

Equations (4) and (11) describe the effect of the common signal (scenario 3) on power and coherence respectively. Unlike power and coherence, there are currently no analytical results describing how a common signal affects instantaneous interaction and total Granger causality. However, we note that if we know the effect of a common signal on the spectral density matrix, then we can use the non-parametric estimation procedure to obtain the affected transfer function and noise covariance matrix.

To see the effect of the common signal on the spectral density matrix we re-write scenario 3 in the frequency domain as

$$\begin{array}{l} \left(\begin{array}{c} Y_{1}\left(\omega \right)\\ Y_{2}\left(\omega \right) \end{array}\right)=\left(\begin{array}{c} X_{1}\left(\omega \right)\\ X_{2}\left(\omega \right) \end{array}\right)+\left(\begin{array}{c} U\left(\omega \right)\\ U\left(\omega \right) \end{array}\right)\\ =\left(\begin{array}{c} H_{11}\left(\omega \right)H_{12}\left(\omega \right)\\ H_{21}\left(\omega \right)H_{22}\left(\omega \right) \end{array}\right)\left(\begin{array}{c} \varepsilon \left(\omega \right)\\ \eta \left(\omega \right) \end{array}\right)+\left(\begin{array}{c} U\left(\omega \right)\\ U\left(\omega \right) \end{array}\right) \end{array}$$

Next we can calculate the spectral density matrix by multiplying both side by the conjugate transpose

(40) $$\begin{array}{l} \left(\begin{array}{c} Y_{1}(\omega )Y_{1}^{*}(\omega )Y_{1}(\omega )Y_{2}^{*}(\omega )\\ Y_{2}(\omega )Y_{1}^{*}(\omega )Y_{2}(\omega )Y_{2}^{*}(\omega ) \end{array}\right)=(\left(\begin{array}{c} H_{11}(\omega )H_{12}(\omega )\\ H_{21}(\omega )H_{22}(\omega ) \end{array}\right)\left(\begin{array}{c} \varepsilon (\omega )\\ \eta (\omega ) \end{array}\right)\\ \;+\left(\begin{array}{c} U(\omega )\\ U(\omega ) \end{array}\right))(\left(\begin{array}{c} H_{11}(\omega )H_{12}(\omega )\\ H_{21}(\omega )H_{22}(\omega ) \end{array}\right)\times {\left(\begin{array}{c} \varepsilon (\omega )\\ \eta (\omega ) \end{array}\right)+\left(\begin{array}{c} U(\omega )\\ U(\omega ) \end{array}\right))}^{*}\\ \;=\left(\begin{array}{c} H_{11}(\omega )H_{12}(\omega )\\ H_{21}(\omega )H_{22}(\omega ) \end{array}\right)\left(\begin{array}{c} \varepsilon (\omega )\\ \eta (\omega ) \end{array}\right){\left(\begin{array}{c} \varepsilon (\omega )\\ \eta (\omega ) \end{array}\right)}^{*}\times {\left(\begin{array}{c} H_{11}(\omega )H_{12}(\omega )\\ H_{21}(\omega )H_{22}(\omega ) \end{array}\right)}^{*}\\ \;+\left(\begin{array}{c} U(\omega )\\ U(\omega ) \end{array}\right){\left(\begin{array}{c} U(\omega )\\ U(\omega ) \end{array}\right)}^{*}=Q(\omega )+\left(\begin{array}{c} U(\omega )U^{*}(\omega )U(\omega )U^{*}(\omega )\\ U(\omega )U^{*}(\omega )U(\omega )U^{*}(\omega ) \end{array}\right) \end{array}$$

Note that the cross-spectra between the common signal [*U*(ω)] and any other quantity [e.g., *H*(ω), ε(ω), η(ω)] vanish since we assumed independence between them. Thus, to obtain the spectral density matrix [**Q**^*C*^(ω)] of the unidirectionally-connected system with common signal, we simply add the power of the common signal [*U*(ω)U^*^(ω)] to the spectral density matrix of the unidirectionally-connected system without common signal [**Q**(ω)]. Once the affected spectral density matrix is known, total Granger causality and instantaneous interaction are derived as before.

#### Simulation for the disconnected system (scenarios 2 and 4)

For the disconnected system (scenario 2) we followed a similar procedure but used a modified spectral density matrix $Q^{\prime }\left(\omega \right)=\left(\begin{array}{cc} s_{11}\left(\omega \right) & 0\\ 0 & s_{22}\left(\omega \right) \end{array}\right)$. This spectral density matrix describes a disconnected system with identical power spectra to the unidirectionally-connected system. Coherence, instantaneous interaction and total Granger causality are all zero for the disconnected system when there is no common signal.

The effect of the common signal (scenario 4) on the power spectra is identical to the unidirectionally-connected system (Equation 4). However, the effect on coherence is now given by Equation (12). Further, we can estimate the Neural signal to Common signal Ratio (NCR) from coherence using Equation (14).

The effect of the common signal on instantaneous interaction and total Granger causality is obtained as for the unidirectionally-connected system, but using **Q**′(ω) instead of **Q**(ω).

We note that an alternative simulation approach would be to use the auto-regressive descriptions to generate time domain data with or without a common signal, and then analyze coherence, total Granger causality and instantaneous interaction in these data (e.g., Ding et al., 2006; Wen et al., 2013). If a large enough amount of data is used then the two approaches yield identical results (which we also verified in simulation). However, simulating time domain data involves more parameters. At the very least we would need to decide on the amount of data (number of trials/length of trials/sampling rate). We would also need to describe the frequency domain analysis, which can include parameters such as the number of tapers for multitaper analysis. Another drawback is that these simulations will suffer from empirical biases when using finite data, which are known to affect coherence (Jarvis and Mitra, 2001) and Granger causality (Barrett et al., 2012). While all of these are important aspects that deserve further consideration, we feel that they would unnecessarily complicate our simulation procedure. They may also make interpretation more difficult, as differences between the scenarios may dependent on the choice of these additional parameters.

#### Experimental setup

To test the utility of the mathematical framework we analyzed previously published data of spontaneous LFPs recorded from the brains of flies. The full experimental details can be found in Cohen et al. (2016, 2018). We briefly recap the relevant details below.

Thirteen female *D. melanogaster* flies were tethered and positioned on an air-supported Styrofoam ball. Linear silicon probes with 16 electrodes separated by 25 μm were inserted laterally to the eye of the fly until the most peripheral electrode site was just outside the eye. This probe covers approximately half of the brain (Figure 1A). A fine tungsten wire was inserted in the thorax and used as a reference electrode (Figure 1B).

#### Data analysis

Data was recorded at 25 kHz and downsampled to 1,000 Hz and the most peripheral electrode site was removed from the analysis. We analyzed both unipolar signals and bipolar derivations. Unipolar signals corresponded to the remaining 15 channels, recorded with respect to a reference electrode located in the flies' thorax (Figure 1B). The bipolar derivations were obtained by subtracting adjacent unipolar signals, providing another set of 14 channels (Figure 1C).

Because we use a linear array recording, the separation between electrodes increases linearly in multiples of 25 μm (Figure 1A). We take the position of the unipolar signals to be the position of the electrode. We take the position of the bipolar derivation to be the mid point of the two unipolar signals used in its derivation. Thus, the separation increases in multiples of 25 μm.

We analyzed 8 consecutive epochs of 2.25 s corresponding to 18 s of spontaneous LFPs (as in Cohen et al., 2018). We removed line noise from each epoch, separately for unipolar or bipolar, using the rmlinesmovingwinc.m function from the Chronux toolbox (http://chronux.org/; Mitra and Bokil, 2007) with three tapers, a window size of 0.75 s and a step size of 0.375 s. We preprocessed each epoch by linearly detrending, followed by subtraction of the mean and division by the standard deviation across time points (i.e., z-scoring across time). Example of the resulting pre-processed unipolar and bipolar LFPs from one fly are shown in Figures 1B,C respectively.

#### Spectral density matrix estimation

We computed the auto- and cross-spectra, $S_{ij}^{e}\left(\omega \right)$, for each unipolar pair *y*_*i*_(*t*) and *y*_*j*_(*t*) (i, j = [1–15]) for each epoch *e* (1–8) over the 2.25 s using the multitaper method based on the MATLAB Chronux toolbox (http://chronux.org/; Mitra and Bokil, 2007) function mtspectrumc.m with 9 tapers, giving a half bandwidth of 2.22 Hz (Mitra and Pesaran, 1999). We obtained the spectral density matrix ${\text{Q}}_{ij}^{e}\left(\omega \right)$ for *y*_*i*_(*t*) and *y*_*j*_(*t*) by setting the diagonal elements to the auto-spectra and the cross-diagonal elements to the cross-spectra (Wen et al., 2013)

$$\begin{array}{l} {\text{Q}}_{ij}^{e}\left(\omega \right)=\left(\begin{array}{cc} S_{ij}^{e}\left(\omega \right) & S_{ji}^{e}\left(\omega \right)\\ S_{ij}^{e}\left(\omega \right) & S_{jj}^{e}\left(\omega \right) \end{array}\right) \end{array}$$

For the final estimate of the unipolar spectral density matrix **Q**_*ij*_(ω), we averaged ${\text{Q}}_{ij}^{e}\left(\omega \right)$ across the 8 epochs (*e* = 1…8). To estimate the bipolar spectral density matrices *b***Q**_*ij*_(ω), we repeated this for the bipolar derivations. Power, coherence, total Granger causality, and instantaneous interaction were estimated from the unipolar and bipolar spectral density matrices, as described below.

#### Power analysis

We obtained an overall estimate of unipolar power by averaging the power spectra across all channels in units of 10log10

$$\begin{array}{l} S\left(\omega \right)=\frac{1}{15}\overset{15}{\underset{i=1}{\sum }}10\log10\left(S_{ii}\left(\omega \right)\right) \end{array}$$

We obtained analogous estimates of bipolar power *bS*(ω) using

$$\begin{array}{l} bS\left(\omega \right)=\frac{1}{14}\overset{14}{\underset{i=1}{\sum }}10\log10\left(bS_{ii}\left(\omega \right)\right) \end{array}$$

The group average unipolar and bipolar power is reported in Figure 1D.

#### Coherence analysis

Coherence (Equation 9) is directly calculated from the cross-spectral density matrices. As discussed in the main text and Appendix A in Supplementary Materials, the effect of common signals due to volume conduction on coherence may depend on the distance separating the two signals. To assess effects due to the distance, we grouped channel pairs into 4 groups: long- (175–325 μm), mid- (75–150 μm), short-distance (50 μm), and adjacent pairs (25 μm).

Specifically, for the unipolar coherence we report

$$\begin{array}{r} C_{25\mu m}\left(\omega \right)=\frac{1}{14}\overset{14}{\underset{j=1}{\sum }}C_{j,j+1}\left(\omega \right) \end{array}$$

$$\begin{array}{r} C_{50\mu m}\left(\omega \right)=\frac{1}{13}\overset{13}{\underset{j=1}{\sum }}C_{j,j+2}\left(\omega \right) \end{array}$$

(41) $$\begin{array}{l} C_{75-150\mu m}(\omega )=\frac{1}{12+11+10+9}(\sum_{j=1}^{12}C_{j,j+3}(\omega )\\ \;+\sum_{j=1}^{11}C_{j,j+4}(\omega )+\sum_{j=1}^{10}C_{j,j+5}(\omega )\\ \;+\sum_{j=1}^{9}C_{j,j+6}(\omega )) \end{array}$$

$$\begin{array}{l} C_{175-325\mu m}(\omega )=\frac{1}{6+5+4+3+2}(\sum_{j=1}^{6}C_{j,j+9}(\omega )\\ \;+\sum_{j=1}^{5}C_{j,j+10}(\omega )+\sum_{j=1}^{4}C_{j,j+11}(\omega )\\ \;+\sum_{j=1}^{3}C_{j,j+12}(\omega )+\sum_{j=1}^{2}C_{j,j+13}(\omega )) \end{array}$$

Equivalently, for the bipolar coherence we report

(42) $$\begin{array}{r} bC_{25\mu m}\left(\omega \right)=\frac{1}{13}\overset{13}{\underset{j=1}{\sum }}bC_{j,j+1}\left(\omega \right) \end{array}$$

$$\begin{array}{r} bC_{50\mu m}\left(\omega \right)=\frac{1}{12}\overset{12}{\underset{j=1}{\sum }}bC_{j,j+2}\left(\omega \right) \end{array}$$

$$\begin{array}{l} bC_{75-150\mu m}(\omega )=\frac{1}{11+10+9+8}(\sum_{j=1}^{11}bC_{j,j+3}(\omega )\\ \;+\sum_{j=1}^{10}bC_{j,j+4}(\omega )+\sum_{j=1}^{9}bC_{j,j+5}(\omega )\\ \;+\sum_{j=1}^{8}bC_{j,j+6}(\omega )) \end{array}$$

$$\begin{array}{l} bC_{175-325\mu m}(\omega )=\frac{1}{5+4+3+2+1}(\sum_{j=1}^{5}bC_{j,j+9}(\omega )\\ \;+\sum_{j=1}^{4}bC_{j,j+10}(\omega )+\sum_{j=1}^{3}bC_{j,j+11}(\omega )\\ \;+\sum_{j=1}^{2}bC_{j,j+12}(\omega )+\sum_{j=1}^{1}bC_{j,j+13}(\omega )) \end{array}$$

Examining finer channel groupings did not reveal any additional insights.

#### Granger causality analysis

To calculate total Granger causality and instantaneous interaction we factorized the unipolar (**Q**_*ij*_(ω)) and bipolar (*b***Q**_*ij*_(ω)) spectral density matrices as described in *Non-parametric estimation of spectral Granger causality*. We averaged total Granger causality and instantaneous interaction per channel separation in the same way we did for coherence (Equations 41 and 42).

## Author contributions

DC and NT designed research, wrote the paper. DC analyzed data, performed research.

### Conflict of interest statement

The authors declare that the research was conducted in the absence of any commercial or financial relationships that could be construed as a potential conflict of interest. The reviewer AS and handling Editor declared their shared affiliation.
