# Supplementary material for: The Effect of Common Signals on Power, Coherence and Granger Causality: Theoretical Review, Simulations, and Empirical Analysis of Fruit Fly LFPs Data
Source: Front Syst Neurosci. 2018 Jul 25;12:30. doi: 10.3389/fnsys.2018.00030 (PMC6068358; doi:10.3389/fnsys.2018.00030)
Supplement: Supplementary file 1 [file Data_Sheet_1.docx]

**Appendix A: Incorporating common signals due to volume conduction into the mathematical framework**

Under our assumptions that 1) electrical activity at the reference electrode is independent of the neural activity and 2) that the neural activity is independent in high frequencies the expression for coherence from Eq. reduces to

$$\frac{abs(X_{i}(\omega_{h})VC_{j}^{*}(\omega_{h})+X_{j}(\omega)VC_{i}^{*}(\omega_{h})+VC_{i}(\omega_{h})VC_{j}^{*}(\omega_{h})+R(\omega_{h})R^{*}(\omega_{h}))^{2}}{(X_{i}(\omega_{h})X_{i}^{*}(\omega_{h})+2\mathrm{Re}(X_{i}(\omega_{h})VC_{i}^{*}(\omega_{h}))+VC_{i}(\omega_{h})VC_{i}^{*}(\omega_{h})+2\mathrm{Re}(VC_{i}(\omega_{h})R^{*}(\omega_{h}))+R(\omega_{h})R^{*}(\omega_{h}))(X_{J}(\omega_{h})X_{J}^{*}(\omega_{h})+2\mathrm{Re}(X_{J}(\omega_{h})VC_{J}^{*}(\omega_{h}))+VC_{J}(\omega_{h})VC_{J}^{*}(\omega_{h})+2\mathrm{Re}(VC_{J}(\omega_{h})R^{*}(\omega_{h}))+R(\omega_{h})R^{*}(\omega_{h}))}$$

which is considerably more complicated then our previous expression for coherence under these assumptions, which has three terms in total (Eq. ). To make further progress we could further assume that for high frequencies the common signals due to volume conduction are also independent of the neural activity. In this case coherence reduces to

$$C_{ij}(\omega_{h})=\frac{abs(VC_{i}(\omega_{h})VC_{j}^{*}(\omega_{h})+R(\omega_{h})R^{*}(\omega_{h}))^{2}}{(X_{i}(\omega_{h})X_{i}^{*}(\omega_{h})+VC_{i}(\omega_{h})VC_{i}^{*}(\omega_{h})+R(\omega_{h})R(\omega_{h}))(X_{j}(\omega_{h})X_{j}^{*}(\omega_{h})+VC_{j}(\omega_{h})VC_{j}^{*}(\omega_{h})+R(\omega_{h})R(\omega_{h}))^{*}}$$

Because the term $VC_{i}(\omega_{h})VC_{j}^{*}(\omega_{h})$ is specific for each electrode pair, it can capture distance dependent effects. In general, we expect that the closer $i$ and $j$are, the larger the contribution of $VC_{i}(\omega_{h})VC_{j}^{*}(\omega_{h})$ to $abs(VC_{i}(\omega_{h})VC_{j}^{*}(\omega_{h})+R(\omega_{h})R^{*}(\omega_{h}))^{2}$ is. Note, however, that changes in the power of the signals (the denominator of Eq. ) also have to be taken into account when attributing any distance dependent effects to the numerator. In our previous expression for unipolar coherence in high frequencies (Eq. ) the numerator equaled the power of the common signal, so there could not be any dependency on the distance separating the electrodes. The point that we wish to make with respect to our analysis of fly LFPs, is that if we observe a reduction in coherence in high frequencies with increasing separation between the electrodes, we may tentatively infer that the common signals involve volume conduction.

For completeness, we note that by making further assumptions we can obtain yet a simpler expression for coherence that still incorporates a distance dependent effect. Specifically, we assume that the power of the common signals due to volume conduction is equally reflected at each electrodes;$VC_{i}(\omega_{h})VC_{i}^{*}(\omega_{h})=VC_{j}(\omega_{h})VC_{j}^{*}(\omega_{h})=VC(\omega_{h})VC^{*}(\omega_{h})$, and that the corresponding cross-spectrum can be modeled as a contribution inversely proportional to the distance separating the two electrodes; $VC_{i}(\omega_{h})VC_{j}^{*}(\omega_{h})=\frac{a}{|i-j|}$, where $a$ is a scalar. The resulting expression for coherence is

$$C_{ij}(\omega_{h})=\frac{abs(\frac{a}{abs(i-j)}+R(\omega_{h})R^{*}(\omega_{h}))^{2}}{(X_{i}(\omega_{h})X_{i}^{*}(\omega_{h})+VC(\omega_{h})VC^{*}(\omega_{h})+R(\omega_{h})R^{*}(\omega_{h}))(X_{j}(\omega_{h})X_{j}^{*}(\omega_{h})+VC(\omega_{h})VC^{*}(\omega_{h})+R(\omega_{h})R^{*}(\omega_{h}))}$$

It is important to emphasize that bipolar derivations can remove common signals irrespective of whether these are due to electrical activity at the reference or due to volume conduction from other electrical sources. The required assumption is only that for *nearby* unipolar signals the common signals are identical. To see this, rewrite the expression for the bipolar derivations (Eq. ) using the extended expression for the unipolar signals (Eq. )

$$by_{i}(t)=(x_{i}(t)+vc_{i}(t)-r(t))-(x_{i+1}(t)+vc_{i+1}(t)-r(t))$$

The assumption that for nearby unipolar signals the common signals are identical means that $vc_{i}(t)-r(t)=vc_{i+1}(t)-r(t)$, giving $by_{i}(t)=x_{i}(t)-x_{i+1}(t)$, as before.

**Appendix B: Coherence simulations with 1/f-like common signal**

We used coherence simulations (Figure 2) to illustrate how the mathematical framework we presented may manifest in empirical analysis. These simulations highlighted that the effects of a common signal on coherence can be dependent on the specifics of the system in question. In these simulations we considered the common signal to be uncorrelated white noise. The spectrum of such a signal has equal power in all frequencies (i.e. a uniform spectrum). This simple choice for the common signal was sufficient for our illustrative simulations. However, in practice physiological signals can have a different type of spectrum. For example, physiological signals often have a spectrum that decreases with increasing frequency in a 1/f-like manner.

To see how such a common signal may affect coherence we set the spectrum of the common signal to the average power of the two simulated neural signals (UU = (Y1 Y1 + Y2 Y2)/2), since these had 1/f-like spectra. We then re-ran the simulation for the four scenarios as before. The results are shown in Figure S1. Aside from the common signal, the simulations were identical to those reported in the main text (see Coherence simulations in Methods for details).

Since the disconnected systems (Scenarios 1 and 2) do not involve the common signal, the results of these simulations are unaffected (i.e. Figure S1e, f, i and j are identical to Figure 2e,f,i and j). The effect of the common signal on power correspond to an increase equal to the magnitude of the common signal. For the uniform-spectrum common signal the increase in power is equal across all frequencies (Figure 2 g and h), whereas for the 1/f-like common signal the magnitude of the increase decreases as frequency increases, following the 1/f-like shape (Figure 1S g and h).

The effect of the 1/f-like common signal on coherence for the connected system (Figure S1k, described by Eq. ) is qualitatively similar to the effect of the uniform-spectrum common signal (Figure 2k) - coherence values increase overall but still decay with frequency.

The effect of the 1/f-like common signal on coherence for the disconnected system (Figure S1 l , described by Eq. ) appears qualitatively different to the effect of the uniform-spectrum common signal. Whereas for the uniform-spectrum common signal coherence increased with frequency (Figure 2l), for the 1/f-like common signal coherence appears as a near-horizontal line at C = 0.25. The curve actually displays a very gentle downward slope from a maximum of 0.2505 at f=0 to a minimum of 0.2502 at the maximal frequency.

Note that this result is clearly explained by the theoretical framework. As before, Eq. dictates that coherence is related to Neural Signal to Common signal Ratios (NCRs). For the uniform-spectrum common signal the NCRs decreased with frequency (Figure 2m), whereas for the 1/f-like common signal the NCRs remain roughly around 1 for all frequencies. For NCR=1 Eq. gives C=0.25 for all frequencies. Thus, we see that the theoretical framework explains the effects of both the uniform-spectrum and 1/f-like common signals.

This additional simulation helps clarify that the effects of the common signal on coherence are dependent on the spectral characteristics of the system and thus need to be evaluated on a case by case basis. Importantly, however, in both types of the common signal, we observed that the common signal 1) introduces above-zero coherence even for the disconnected system and 2) can lead to an overestimation of coherence for both the connected and disconnected systems (Figure 2 and S1, panels k and l).

Finally, we note that for the empirical analysis *we did not assume* any form for the spectrum of the common signal. Instead we simply investigate coherence in both the unipolar and bipolar data. Having observed that 1) high frequency coherence is very high in the unipolar data and 2) that it is near zero in the bipolar data is *consistent* with there being common signals in the unipolar data (based on our theoretical review and simulations), and that these common signals are largely removed by bipolar referencing.


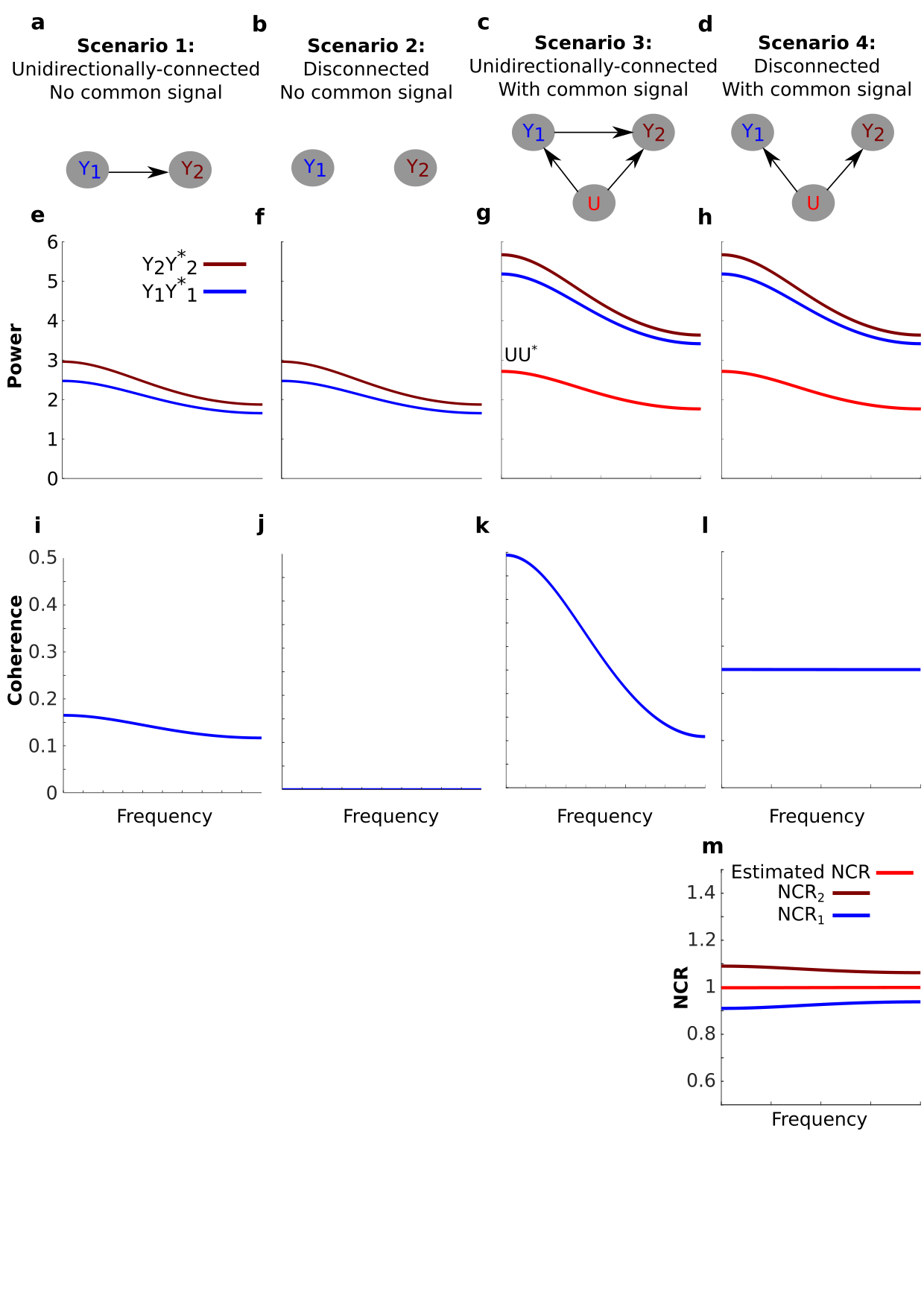


**Figure 1S. The effect of a 1/f-like common signal on coherence. The figure is analogues to Figure 2 but using a common signal with a 1/f-like spectrum instead of a common signal with a uniform spectrum.**

(**a-d**) Schematics of the four simulated scenarios. Since scenarios 1 and 2 do not involve the common signal, panels e, f, i and j are identical to those in Figure 2. The effect of a 1/f-like common signal on power (**g-h**) is an increase equal to the power of the common signal (UU*, 1/f-like common signal). The effect of the 1/f-like common signal on the unidirectionally-connected system. Coherence increases overall but decays with frequency (**k**). For the disconnected system, the 1/f-like common signal results in coherence values around 0.25 for all frequencies (**l**). As before, this result is explained by the Neural to Common signal Ratio (NCR, Eq. ). (**m**) NCR1 (blue) and NCR2 (brown) refer to the NCR of nodes one and two respectively. Because for this system the NCR1~NCR2~1, Eq. gives that C~0.25 for all frequencies. From the observed coherence in **l**, we can also estimate the NCR using Eq. . The estimated NCR is shown in red, which falls between the value of NCR1 and NCR2.
